# Natural antibody response to *Plasmodium falciparum* merozoite antigens MSP5, MSP9 and EBA175 is associated to clinical protection in the Brazilian Amazon

**DOI:** 10.1186/1471-2334-13-608

**Published:** 2013-12-28

**Authors:** Márcia M Medeiros, Wesley L Fotoran, Rosimeire C dalla Martha, Tony H Katsuragawa, Luiz Hildebrando Pereira da Silva, Gerhard Wunderlich

**Affiliations:** 1 Department of Parasitology, Institute of Biomedical Sciences, University of São Paulo, São Paulo, Brazil; 2 Institute for Research in Tropical Pathologies-IPEPATRO, Porto Velho, Rondônia, Brasil

## Abstract

**Background:**

Antibodies have an essential role in the acquired immune response against blood stage *P. falciparum* infection. Although several antigens have been identified as important antibody targets, it is still elusive which antigens have to be recognized for clinical protection. Herein, we analyzed antibodies from plasmas from symptomatic or asymptomatic individuals living in the same geographic area in the Western Amazon, measuring their recognition of multiple merozoite antigens.

**Methods:**

Specific fragments of genes encoding merozoite proteins AMA1 and members of MSP and EBL families from circulating *P. falciparum* field isolates present in asymptomatic and symptomatic patients were amplified by PCR. After cloning and expression of different versions of the antigens as recombinant GST-fusion peptides, we tested the reactivity of patients’ plasmas by ELISA and the presence of IgG subclasses in the most reactive plasmas.

**Results:**

11 out of 24 recombinant antigens were recognized by plasmas from either symptomatic or asymptomatic infections. Antibodies to MSP9 (X^2^_DF=1_ = 9.26/*p* = 0.0047) and MSP5 (X^2^_DF=1_ = 8.29/*p* = 0.0069) were more prevalent in asymptomatic individuals whereas the opposite was observed for MSP1 block 2-MAD20 (X^2^_DF=1_ = 6.41/*p* = 0.0206, Fisher’s exact test). Plasmas from asymptomatic individuals reacted more intensely against MSP4 (U = 210.5, *p* < 0.03), MSP5 (U = 212, *p* < 0.004), MSP9 (U = 189.5, *p* < 0.002) and EBA175 (U = 197, p < 0.014, Mann-Whitney’s U test). IgG1 and IgG3 were predominant for all antigens, but some patients also presented with IgG2 and IgG4. The recognition of MSP5 (OR = 0.112, IC_95%_ = 0.021-0.585) and MSP9 (OR = 0.125, IC_95%_ = 0.030-0.529, cross tab analysis) predicted 8.9 and 8 times less chances, respectively, to present symptoms. Higher antibody levels against MSP5 and EBA175 were associated by odds ratios of 9.4 (IC_95%_ = 1.29-69.25) and 5.7 (IC_95%_ = 1.12-29.62, logistic regression), respectively, with an asymptomatic status.

**Conclusions:**

Merozoite antigens were targets of cytophilic antibodies and antibodies against MSP5, MSP9 and EBA175 were independently associated with decreased symptoms.

## Background

Malaria along with HIV/AIDS and Tuberculosis is one of the major causes of mortality and morbidity in tropical areas and consequently the primary obstacle to social and economic improvement for developing countries in these regions [1]. Despite recent efforts to control infections based on the use of insecticide-treated bed nets alone or combined with indoor residual spraying [2] and artemisinin-combined treatment [3], almost 300 million cases and 800,000 deaths are annually registered [1,3]. Most of these deaths occur in Sub-Saharan Africa in children under 5 years and pregnant women as a consequence of *P. falciparum* infection [3]. Given the looming resistance of the parasite against the derivatives of artemisinin, concentrated and continuous efforts are necessary to contain the disease. These include the facilitated access to effective treatment, the introduction of novel drugs and also the development of efficient vaccines.

Important success has been achieved in the development of vaccines based on pre-erythrocytic targets using the circumsporozoite protein [4] or liver stage targets employing knockout parasite lines [5]. The finding that the passive transfer of immunoglobulins led to the suppression of parasite multiplication and temporary cure [6] supports the view that relevant targets are also found on blood stage parasites. Proteins on the infected red blood cell which are mostly variant and with relatively low immunogenicity can be targets of antibodies which recruit the infected cells for phagocytosis. Proteins on the merozoite actively participate in the successive coordinated events that culminate in the erythrocyte invasion [7]. Several merozoite surface proteins and others secreted by the apical organelles interact with erythrocyte ligands. This interaction favors adhesion, apical reorientation and creation of a moving junction that allows the invagination of this parasite form into the erythrocyte, leading to the formation of a parasitophorous vesicle membrane (PVM) in which the parasite resides after invasion (reviewed in [8]). Many of the merozoite antigens act as targets of the natural antibody immune response [9,10] and several of them have been implicated in the development of clinical protection [11-17] and therefore are present in the anti-blood stage vaccine formulations which are currently being tested [18-21].

In the Brazilian Amazon, the transmission and incidence of *P. falciparum* malaria is quite different from Africa [22]. Many localities which experienced periods of high malaria transmission in the past [23], presently show low and seasonal *P. falciparum* transmission [24]. There is also a significant genetic structuring in parasites from the Western Amazon suggesting a paucity of different circulating strains [25,26]. Coincidently, the repertoire of variant genes of *P. falciparum* is restricted and redundant [27]. This special situation of exposure to restricted numbers of antigens in addition to the observation of uncomplicated infections probably explains previous data regarding a high incidence of asymptomatic carriers and/or persons to which the majority of new infections are without symptoms [28]. Regardless of the apparently functional immune protection of these asymptomatic carriers, they do represent a persistent source of *P. falciparum* infections [29] in Amazonian settings.

On the basis of this epidemiologic background characterized as a high incidence of asymptomatic infections with the occurrence of sporadic symptomatic cases in the same population in the Amazon, we set out to analyze which parameter of the humoral immune response against merozoite antigens is decisive for the observed outcome during a *P. falciparum* malaria infection. To do this, we focused on the recognition of a number of antigens involved in the erythrocyte invasion process, namely proteins of the MSP and EBL families and AMA1. In order to test the response against relevant versions of target proteins, we analyzed the circulating alleles of merozoite genes from field isolates present in the blood of sympatric symptomatic and asymptomatic carriers and produced parts of them as recombinant antigens. Then, the humoral immune response against these antigens was measured by ELISA and correlated to disease outcome and epidemiological parameters.

## Methods

### Study site

The study was conducted in a riverside area of Porto Velho, the capital of Rondônia state, in the Western Brazilian Amazon. Four localities on the riverbanks of the Madeira River were chosen, Vila Candelaria, Bate-Estaca, Santo Antonio and Engenho Velho. The first three are located one after the other on the right bank of the Madeira River and the last, on the left bank. As in other endemic settings in Brazil, the majority of malaria cases in these areas are caused by *Plasmodium vivax*[22] and the overall endemicity for Malaria is relatively low*.* The annual distribution of malaria cases in these areas paralleled the seasonal rain distribution with a peak of incidence in the beginning (October-November) and other after the peak of rainy season (February-March) [24]. The principal vector in Brazilian malaria endemic areas is *Anopheles darlingi*[22]*.*

### Study design

During the low malaria transmission season (August-September), a cross sectional study was conducted in the four localities by the IPEPATRO field team and FUNASA technicians. Children older than one year to seniors, both male and female, were invited to participate and more than 50% of residents from each locality agreed to participate. After each participant (or the legal guardian for children) signed a specific consent form, a 5 ml blood sample was collected by peripheral venipuncture, independent of the presence of malaria symptoms. Individuals with any malaria symptoms (fever >37.8°C, prostration, headache, chills, tremors, myalgia, nausea and vomiting) and *P. falciparum* positive thick blood films were immediately treated after blood was taken and were included in the symptomatic group. The *P. vivax* symptomatic patients were immediately treated and were not included in the study. The participants without symptoms but positive by PCR for *P. falciparum* were followed up for 1 month by a medical team to verify the development of symptoms. When symptoms appeared, a thick blood film was performed and individuals which were positive for falciparum malaria were treated immediately and included in the symptomatic group. Persons who did not develop symptoms even with *P. falciparum* positive PCR were classified asymptomatic and treated after the follow-up. Persons with positive PCR for *P. vivax* without symptoms were treated immediately after the parasite diagnosis and not included in the study. Only one asymptomatic patient presented parasites in the thick blood smear (routinely, 50 fields per blood smear were monitored) while the remaining asymptomatic infections were only diagnosed by PCR. Finally, 28 samples were obtained from symptomatic patients (28 erythrocyte concentrates and 27 plasma samples) and 25 from asymptomatic patients (25 erythrocyte concentrates and 24 plasma samples). In order to calculate reactivity indices, 13 plasma samples from persons living in non-endemic regions of Brazil and never exposed to malaria were included as the negative control group.

### Ethical statement

The study was approved by the Ethics Committee for Research involving Humans at the Institute of Biomedical Sciences of University of São Paulo (protocol 741/2006). The patients were treated according to the guidelines for treating malaria patients of Brazil’s Health Ministry, available at the site (http://bvsms.saude.gov.br/bvs/publicacoes/guia_pratico_malaria.pdf) based on WHO recommendations (http://whqlibdoc.who.int/publications/2010/9789241547925_eng.pdf).

### Blood sampling

The blood samples were collected in sterile tubes with EDTA, centrifuged (400 g, 5 minutes, room temperature) and fractioned into plasma and packed erythrocytes. One volume of glycerol was added to each plasma sample and these samples were maintained at −80°C. 250 μl of the packed erythrocytes were submitted to *Plasmodium* genomic DNA (gDNA) purification by the Proteinase K/phenol-chloroform method [30].

### Identification of *Plasmodium* infection by PCR, locus amplification

Each gDNA extracted was submitted to nested PCR in order to verify the presence of the *Plasmodium* genus by amplification of ssrRNA fragments as described previously [30]. According to the PlasmoDB database (v5.5), primer pairs covering polymorphic regions and/or immune epitopes of MSP4, MSP5, MSP6, MSP7, MSP9, MSP10, AMA1, EBA140, EBA175 and EBA181 were designed on the basis of the 3D7 genome sequences. Primers described previously to amplify MSP1 block 2 [31], the polymorphic regions of MSP2 [32] and MSP3 [11] and the M2 domain of MAEBL [33] were used. A *Bam*HI or BglII restriction site was added at the 5′ extremity of each forward oligonucleotide. The sequences of the primer pairs used as well as the amplified regions are shown in Additional file 1: Table S2 and in Additional file 2: Figure S2, respectively. Field isolate gDNAs extracted from blood from symptomatic and asymptomatic infections were submitted to 35 amplification cycles of denaturation at 94°C (1 minute), annealing at temperatures specific for each pair of primers (1 minute), polymerization at 72°C (1 minute) and a final polymerization at 72°C for 10 minutes. Taq Polymerase enzyme (Fermentas) was used according to the manufacturer’s instructions. In all reactions, 3D7 gDNA was used as a positive control and an amplification solution without gDNA as negative control.

### Cloning and sequence analysis

All amplified fragments from each gDNA were A/T cloned in pGEM-T easy vector (Promega) according to the manufacturer’s instructions. Ligations were transformed in *E. coli* DH10B cells. Five recombinant clones were analyzed by automated DNA sequencing using the BigDye 3.1 Terminator Cycle Sequencing kit (Applied Biosystems) according to the manufacturer’s instructions and sequencing was conducted in an ABI 3100 automatic sequencer (Applied Biosystems). The identity of each sequence was confirmed by BlastN analysis. All obtained *P. falciparum* sequences of each merozoite gene were loaded in *Clustal X* (1.83) and aligned. An identity matrix was generated for each sequence group and sequences were clustered into groups with identities of 95% or more which were then considered identical. This number was chosen based on the assumption that differences above this cutoff would most probably generate proteins which are recognized similarly by antibodies present in human sera. One representative sequence from each group of >95% identical sequences was subcloned via BamH1/EcoR1 digestion and ligated in pGEX2T (Amersham Pharmacia). Data regarding the sequence diversity of MSP1 block 2 [34] and MSP2 [35] were used to define allelic groups and variants.

### Protein expression

Recombinant pGEX2T plasmids were transformed into *E. coli* BL21 DE3 pLys Codon Plus RIL cells and grown on a shaker at 37°C in 5 ml aliquots overnight in LB supplemented with ampicillin (100 μg/μl) and chloramphenicol (34 μg/μl). 1 ml of the preinoculum was then transferred into 100 ml LB supplemented with ampicillin (100 μg/μl) and grown to optical densities (600 nm) of ≥ 0,6 and induced by the addition of IPTG to a final concentration of 0,1 mM for 3 hours at 37°C on a shaker at 200 rpm. Bacterial cultures were then centrifuged for 20 minutes at 4°C and 2300 g in a Sorvall R7 benchtop centrifuge. The bacterial pellet was dissolved in 5 ml PBS/1% Triton X-100 (pH 7.4) and Lysozyme (0,1 mg/ml, Sigma) was added. The solution was then incubated rocking for 5 minutes at room temperature and frozen for 2 hours or overnight at −20°C. Afterwards, the material was thawed and submitted to five sonication cycles (Branson sonifier, 30 seconds, force 40, on ice) intercalated by 30 seconds without sonication. 5 ml of PBS 1X/1% Triton X-100 was added and centrifuged as described above. Supernatants were then incubated with 100–200 μl of Glutathione-sepharose resin (Amersham Pharmacia) pre-washed with PBS (pH 7.4), under continuous agitation at room temperature. After one hour, the resin was washed once with PBS/1% Triton X-100 and twice with PBS. Then, 500–750 μl of elution buffer (0,1 M Tris–HCl pH 8, 0,12 M NaCl and 10 mM reduced gluthatione) were added to the resin in 1,5 ml micro tubes, and incubated at RT under continuous agitation for 1 hour, after which the supernatant was harvested by centrifugation for 1 minute, 12000 rpm. Recombinant proteins were submitted to electrophoresis in SDS-PAGE under reducing conditions. All expressed peptides were submitted to Western Blotting analyses with anti-GST mouse serum to test the correct size of the fusion protein (data not shown). The concentration of recombinant proteins was calculated by the Bradford method.

### ELISA assays

Flat wells plates (medium binding) were coated overnight at 4°C with 50 μl polypeptide solution (125 ng/well) in sodium carbonate buffer (50 mM, pH 9.6). After one wash with PBS/0.05% Tween 20 (washing solution), 200 μl/well of blocking solution (PBS, 0.05% Tween 20 and 4% skimmed milk) was added for 2 hours at 37°C. Plates were washed three times with washing solution and incubated for one hour at 37°C using 1/200 diluted plasmas from asymptomatic and symptomatic patients. This incubation occurred in blocking solution with 1% skimmed milk (incubation solution). All plasmas were tested in duplicates in wells coated with fusion peptide and background binding to the GST fusion was estimated by wells which contained only rGST. After five washes, plates were incubated with 1/1500 diluted goat anti-human IgG HRP antibodies (KPL) in incubation solution for one hour at 37°C. After five washes, 50 μl/well of TMB substrate (KPL) was added and 10 minutes later the reaction was stopped with 50 μl/well of 1 M HCl. The OD_450nm_ was read in a spectrophotometer using the OD_595nm_ value as reference. Thirteen plasmas from persons without previous contact with *Plasmodium sp*. were used as negative controls. IgG subclass-specific ELISA assays for each peptide were performed as follows: Monoclonal mice antibodies against each anti-human IgG subclass (anti-IgG1 (2C11, ab1927), anti-IgG2 (3C7, ab1935), anti-IgG3 (5G12, ab1928) e anti-IgG4 (5C7, ab1930) (Abcam, Inc., Cambridge, Massachusetts, USA) were added after the primary incubation, diluted 1/3000 in incubation solution. Afterwards, goat anti-mouse IgG HRP antibodies (KPL) were added, 1/2500 in incubation solution.

### Statistical analyses

Microsoft Excel 2007 spreadsheets were used to collect the raw data and perform OD cutoff calculations while statistical analyses were run in SPSS (15.0). The quantitative variables for the epidemiologic data were: age, time living in an endemic malaria area (TLEA), time living at the same actual address (TLSA), number of previous symptomatic falciparum malaria infections (NPMF), time since the last symptomatic malaria falciparum infection (TLMF), number of recombinant antigens recognized in the ELISA assays (NRAR). These variables were compared between symptomatic and asymptomatic groups by Mann-Whitney’s U test for comparisons between two independent samples. *p* values lower than 0.05 were considered significant.

Instead of optical densities, we used reactivity indices (RIs) to estimate the specific antibody reaction, thereby eliminating signals from residual *E. coli* derived components in the recombinant protein preparations. For this, the optical density (OD) values from the negative plasma control group were used to estimate the reaction cut-off for each antigen tested. The average OD_final_ (OD_final_ = OD_antigen_-OD_GST_) plus 2 SD (standard deviations) was taken as the cutoff value. OD_antigen_ is the optical density that was measured using all plasmas on the GST-fused antigen while OD_GST_ is the optical density that was measured the same plasmas on GST-coated wells. The RI for each reactive plasma and each antigen (OD > cutoff) was calculated by dividing the OD value by the cut-off reaction value. The RIs from symptomatic and asymptomatic plasmas for each tested antigen were compared by Mann-Whitney’s U test for comparisons between two independent samples. For comparisons between different alleles or variants, the RIs from each clinical group were compared by Wilcoxon signed rank test for comparisons between paired samples. *p* values lower than 0.05 were considered significant for the two tests. The OD values obtained in IgG subclass ELISA assays were compared between symptomatic and asymptomatic individuals by Mann-Whitney’s U test.

Cross tab analysis was used to evaluate the frequency of reactive plasmas for each antigen in groups of symptomatic and asymptomatic patients. This analysis was also applied to analyze the frequencies of different alleles of polymorphic genes present in parasites collected from these patient groups. The Chi-square (Χ^2^) method or, whenever necessary, Fisher’s exact test identified the differential frequencies (significant when *p* < 0.05).

Association between qualitative dichotomous variables “symptoms” (Yes-symptomatic or No-asymptomatic) and “antigen recognition” (Yes-recognized or No-not recognized) was also verified by cross tab analysis. The strength of association was calculated using the V Cramér coefficient that varies from 0 to 1 (0-no association to 1-strong association). Odds ratio (OR) values were obtained from tabs showing true association among variables, verified by the X^2^ method (*p* < 0.05). OR values represent the ratio of odds of non antigen-recognizing individuals to present an asymptomatic profile in relation to antigen-recognizing individuals. Values lower than 1 indicate that antigen-recognizing individuals are more likely to remain free of symptoms compared to individuals who do not react against tested antigens. Association between the asymptomatic profile and intensity of antigen recognition was verified by logistic regression. The response variable (symptoms) was categorized in 0 (yes = symptomatic) or 1 (no = asymptomatic) and the explanatory variables (RI values) were categorized in terciles. Firstly, each explanatory variable was analyzed by a likelihood ratio test in the univariate model to verify association or not with the response variable. The association force was calculated using the Wald test. The OR points to the ratio of odds (the chances in relation to the non-chances) that each explanatory variable offers for the response variable assuming the “1” value (asymptomatic profile). In the multivariate model, we then verified which explanatory variables were maintained independent in offering chances to present an asymptomatic profile, and this was done by the partial likelihood ratio test.

## Results

### Circulating *P. falciparum* strains in a Western Amazon site show low variability in major merozoite surface antigens

**Figure 1:**
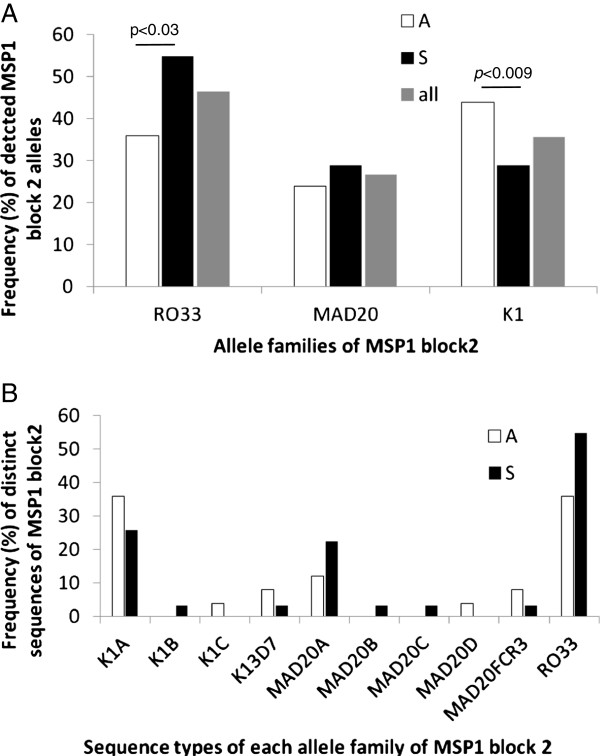
**Frequency of MSP1 block 2 alleles in Rondônia field isolates. A**: RO33 was the most prevalent followed by the K1 allele. The RO33 allele was more prevalent in symptomatic infections while K1 alleles were dominant in asymptomatic samples (Chi-square method, p < 0.03 and p < 0.009, respectively). **B**: K1A and MAD20A were the most frequent K1 and MAD20 type sequences for both groups. Note that the sum of values is higher than 100% since a number of isolates presented more than one genotype at this locus.

The successful immune response against *P. falciparum* malaria is believed to depend on the recognition of both variant antigens on the infected red blood cell and also merozoite antigens. In cases such as allelic forms of MSP1 polymorphic block 2 there is limited cross-recognition between proteins. In order to measure the immune response against antigens which in fact occur in the circulating parasite strains of the infections studied herein, we first identified the sequences of the major antigens. As shown in Additional file 1: Table S1, few different sequences per target were found in the circulating isolates. Due to smaller genomic DNA quantities the amplification success was lower in samples from asymptomatic carriers and the genes from some antigens were not amplified at all in these samples (Additional file 1: Table S1). Importantly, the PCR primer sequences were chosen from alignments of deposited sequences of each gene, avoiding polymorphic regions at the priming sites (Additional file 2: Figure S2). As expected, several different alleles were discovered in the MSP1 polymorphic block 2 fragments in a number of samples indicating polyclonal infections (Additional file 1: Table S1). Most of the non-MSP1 sequences detected were very similar or identical to 3D7 sequences. When deviations from the 3D7 sequences were found, they consisted of point mutations (MSP3 and MAEBL) or variations in the number of repetitions (variants of MSP1 and MSP2 alleles and MSP10). We then wanted to elucidate if the occurrence of certain genotypes was associated with the observed infection type (symptomatic versus asymptomatic). In the case of MSP1 block 2, we observed that the RO33-type sequence was the most frequent allele, present in approximately 46% of all field isolates from the four localities (26/56) (Figure 1). This allele was more frequent (approximately 55%) among isolates collected from symptomatic than asymptomatic infections (X^2^_DF=1_ = 4.178, *p* < 0.03) (Figure 1). K1-type sequences were the most frequent alleles in samples from asymptomatic carriers (44%, X^2^_DF=1_ = 6.676, *p* < 0.009) and K1A, the most frequent variant among this allele family. MAD20-type sequences were present in 24% of isolates from asymptomatic and approximately 29% of isolates from symptomatic infections. Among these, MAD20A was the most frequent over the two clinical groups (Figure 1). Five isolates from asymptomatic infections presented polyclonal infections with two or three different allele sequences. The same was verified in five isolates from symptomatic infections (Additional file 1: Table S1). IC1-type sequences of MSP2 were predominant over the FC27 type in symptomatic infections, however, only one MSP2 sequence was amplified from isolates present in asymptomatic infections, making correlation analysis between infecting genotype and disease outcome impossible, as was the case for the remaining targets (Figure 1 and Additional file 1:Table S1).

### Recognition frequency of recombinant merozoite antigens

In the next step all sequences different at a 95% identity level were expressed as soluble bacterial GST fusion proteins (Additional file 2: Figure S2). In order to assure that the conformation of the produced antigens at least partially reflected native antigens, we heat-inactivated a number of the antigens including rMSP1_19_ and rEBA175 which are known to possess conformational epitopes. As a result, the recognition of MSP1_19_ decreased to half for the plasmas tested, while for EBA175 only a partial decrease was observed (Additional file 2: Figure S3). This indicates that the produced antigens contained conformational and linear epitopes that could be recognized by infection-induced antibodies. Then, the presence of antibodies in plasmas from symptomatic and asymptomatic carriers was tested. Importantly, the decisive factor for inclusion in the “symptomatic” group of individuals was the occurrence of any of the typical malaria symptoms (see Methods section) together with the detection of parasites. On the other hand, the persistent absence of any symptom and the presence of parasites defined the “asymptomatic” individual.

**Figure 2:**
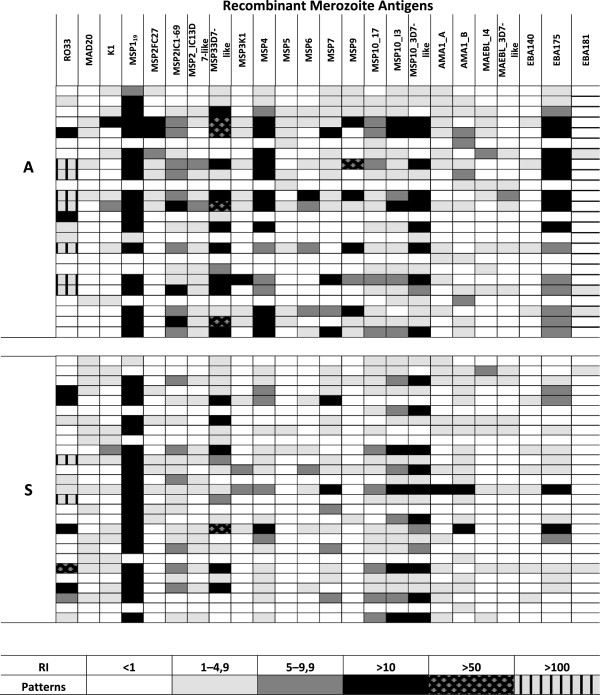
**Antibody recognition of recombinant antigens in symptomatic and asymptomatic patients.** ELISA results computed to reactivity indices are shown and the recognition strength is expressed in color codes. Columns show results for different recombinant proteins being 1-MSP1_RO33, 2-MSP1_MAD20, 3-MSP1_K1, 4-MSP1_19_, 5-MSP2_FC27, 6-MSP2_IC1-69, 7-MSP2_IC1_I4 (3D7-like), 8-MSP3_3D7, 9-MSP3_I4, 10-MSP4, 11-MSP5, 12-MSP6, 13-MSP7, 14-MSP9, 15-MSP10_17, 16-MSP10_I3, 17-MSP10_3D7-like, 18-AMA1_A, 19-AMA1_B, 20-MAEBL_I4, 21-MAEBL_3D7-like, 22-EBA140, 23-EBA175, 24-EBA181. The sequences used for expression are depicted in Additional file 2: Figure S1 and the accession numbers are informed in Additional file 1: Table S1.

**Figure 3:**
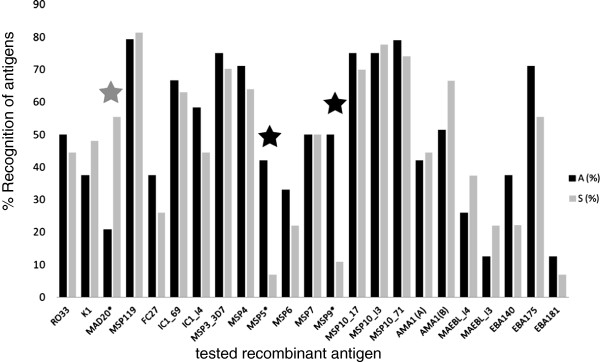
**Percentage of recognition of different proteins by plasmas from asymptomatic (“A”) and symptomatic patients (“S”) from the same area.** The tested antigens are shown on the x-axis while the percentages of plasmas with positive recognition (RI > 1) per clinical group are shown on the y-axis. The reactivity index RI was calculated by dividing OD values from sample plasmas against given antigens by the a media plus 2 standard deviations of OD values obtained with the same antigens recognized by 13 malaria-naïve subjects (see also Methods). Statistical differences are indicated as asterisks and were found for the MAD20-type sequence of MSP1 block 2: S > A (*p* = 0.0206), MSP5: A > S (*p* = 0.0069) and MSP9: A > S (*p* = 0.0047).

We measured the recognition of all antigens by calculating their reactivity indices. This method eliminates the background signal caused by the binding of antibodies against traces of *E. coli* proteins which remained in the preparation of the recombinant antigens. The produced antigens were recognized at different intensities with RI values ranging from 1 to 100 (Figure 2) and the most recognized protein was the C-terminal portion of MSP1, followed by rMSP10, rEBA175 and rMSP3 (Figure 3). On the other hand, other antigens such as rEBA181 and rMAEBL were only recognized by a small fraction of the plasmas. Importantly, all proteins were expressed in a bacterial expression system and the quality of folding is most probably different between different proteins. This permitted only the evaluation of reactions between different plasmas but not any affirmation regarding the absolute IgG quantity against any antigen tested herein or quantitative comparisons between different antigens. We then measured which of these antigens were recognized at a higher frequency by plasmas from asymptomatic or symptomatic patients. As shown in Figure 3, MSP1 block 2 allele MAD20 was recognized by a higher number of symptomatic patients than asymptomatic carriers (*p* = 0.0206, Fisher’s exact test). There was no specific preference of recognition of rMSP1 block 2 alleles by plasmas from individuals infected with a parasite genotype bearing a determined MSP1 block 2 allele (Additional file 2: Figure S4). For example, persons infected with a MSP1 block2-MAD20 type did not have more or less antibodies against RO33 and K1 type alleles, and vice versa. rMSP1_19_, a component of several vaccine formulations, was recognized by equal numbers of individuals from both groups. This was also the case for all other antigens tested herein. Finally, MSP5 and MSP9 were recognized by a higher portion of asymptomatic than symptomatic individuals (MSP5: *p* = 0.0069) and MSP9: *p* = 0.0047, Fisher’s exact test).

### Antibody titers seem to inversely correlate with symptoms

**Figure 4:**
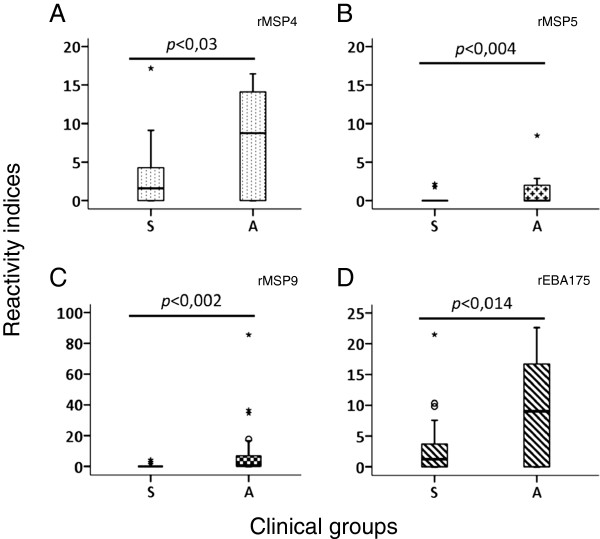
**Significant differences in the recognition of four merozoite antigens by antibodies from asymptomatic individuals.** The distribution of RI values of plasmas recognizing rMSP4 **(A)**, rMSP5 **(B)**, rMSP9 **(C)** and rEBA175 **(D)** among clinical groups (27-Symptomatic (S) and 24-Asymptomatic (A)) is shown. All differences were statically significant (p < 0.05) by Mann-Whitney’s U test.

**Figure 5:**
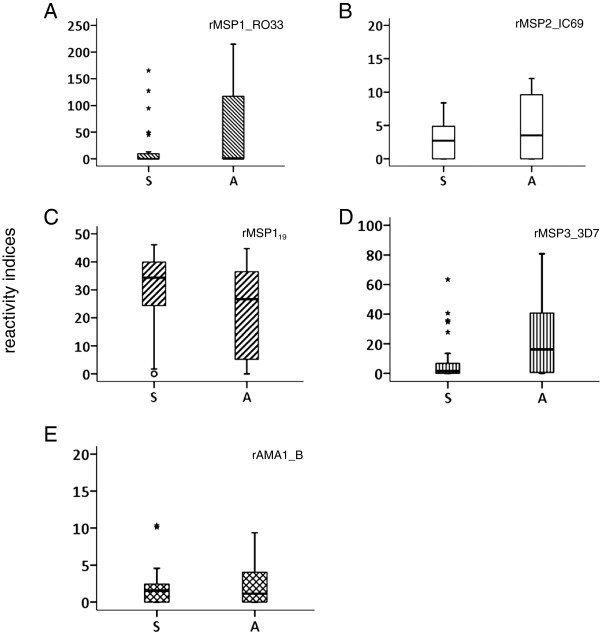
**Relevant vaccine related antigens are not significantly stronger recognized by antibodies from symptomatic or asymptomatic individuals.** Results for the distribution of RI values using plasmas from both clinical groups as in Figure 4 is shown for rMSP1_RO33, rMSP2_IC-69, rMSP1_19_, rMSP3_3D7 and AMA1_B (graphs **A** to **E**, respectively).

The antibody titer and specificity against antigens may be important for lower parasite proliferation rates and indirectly the disease outcome. We therefore evaluated if the quantity of a given antibody was related to the asymptomatic status. For this, we compared the obtained RI values for both clinical groups with the observed disease status at the time of blood withdrawal. Similar to previous studies, very high titers of antibodies, seen as the highest RIs, were observed against parts of the merozoite protein 1 antigens rMSP1_19_ and the allelic forms of MSP1, polymorphic block 2. The protein rMSP3_3D7-like was largely recognized by plasmas from both clinical groups and RI values higher than 10 represented 50% of RI values in the asymptomatic group whereas in the symptomatic group these values represented 18.5% of the RI values. Four plasmas in the asymptomatic group had RIs higher than 50 against rMSP3_3D7-like whereas only one presented with this feature in the symptomatic group. Plasmas also reacted strongly and equally against rMSP10_I3 and rMSP10_3D7-like and showed RI > 10 in both symptomatic and asymptomatic infections. Only plasmas from asymptomatic patients had RI values of more than 10 to antigens rMSP2_FC27, rMSP2_IC1, rMSP4, rMSP9 and rEB175, and some plasmas had an RI > 50 to rMSP9. When tested for significant differences, plasmas from asymptomatic patients had significantly higher RIs against rMSP4 (U = 210.5, *p* < 0.030), rMSP5 (U = 212, *p* < 0.004), rMSP9 (U = 189.5, *p* < 0.002) and rEBA175 (U = 197, *p* < 0.014), as tested by Mann-Whitney’s *U* test (Figure 4) while other strongly recognized antigens showed no statistically significant differences (Figure 5). Importantly, rMSP5 was recognized by 42% of plasmas from the asymptomatic group and the observed RI values were only 1 to 4.9.

### Protection from clinical malaria or development of the asymptomatic profile is independently associated with the recognition of MSP5, MSP9 and EBA175

We performed two types of analyses to investigate the association between the antibody response to a specific antigen and the asymptomatic profile, working with the variable RI as either a qualitative or a quantitative parameter. The crosstab analysis between the qualitative dichotomous variables “symptoms” (Yes = symptomatic and No = asymptomatic) and “antigen recognition” (quantitative variable RI transformed in qualitative variable Yes = recognized (RI > 1) or No = not recognized (RI ≤ 1)) predicted individuals were 8.9 or 8 times less likely to present with symptoms if their plasma recognized rMSP5 (OR = 0.112, IC_95%_ = 0.021-0.585) or MSP9 (OR = 0.125, IC_95%_ = 0.030-0.529) respectively. Using logistic regression analysis, the intensity of the recognition of rMSP5 and rEBA175 (higher RI values against these antigens produced by plasmas from asymptomatic individuals grouped in the 3^rd^ tercile) was independently associated with the explanatory variable “clinical profile-symptomatic or asymptomatic”. Higher antibody levels against rMSP5 and rEBA175 resulted in an odds ratio of 9.4 (IC_95%_ = 1.29-69.25) and 5.7 (IC_95%_ = 1.12-29.62, logistic regression), respectively, to associate with the asymptomatic status (Tables 1, 2 and 3). Notably, not all asymptomatic patients had antibodies against these antigens, but they were recognized more frequently and stronger by asymptomatic individuals compared to symptomatic patients. We also did not find a relevant association between these antigens and any other epidemiological parameter described in Materials and Methods. This seems to point to the hypothesis that the acquisition of protection to clinical malaria probably involves the recognition of other relevant antigens or the development of other important features of the immune response, such as the regulation of the pro-inflammatory immune response.

**Table 1:** **Variables associated with the history of exposure to malaria parasites in general and to *P. falciparum***

**Variables**	**Number of individuals (A/S)**	**Range**	**Median in whole sample**	**25%-75% percentiles whole sample**	**Median from A group**	**25%-75% percentiles from A group**	**Median from S group**	**25%-75% percentiles from S group**
**Age (y)**	51 (24/27)	4-84	24	15-40.5	28	16-53	22	13-34
**TLEA (y)**	51 (24/27)	4-83	22.5	14-40.5	27	15-53	21	13-33
**TLSA (y)**	51 (24/27)	0-60	10	4.75-23.25	10	4-28	9	5-20
**NPEF**	47 (23/24)	0-30	4	2-5	4	1-8	3	2-5
**NAR**	51 (24/27)	0-26	13	7-16	14	6-18	13	7-14
**TLSE (y)**	48 (24/24)	0-30	1	1-3.5	2	1-14	1	1-1

Shown are the parameters symptomatic (S), asymptomatic (A), year (y), time of living in endemic area (TLEA), time of living in the same address (TLSA), number of previous episodes of *falciparum* infection (NPEF), number of recombinant antigens recognized (NAR) and time since the last symptomatic episode (TLSE). Note that some information could not be obtained from the participants leading to varying values in column 2.

**Table 2:** Antigens correlated to a lower risk for symptoms

**Antigens**	***X***^***2***^**/( *p *)**	**V de *Cramér*/(*p*)**	***Odds ratio***	**CI 95% para OR**
**MSP5**	8.288 (0.004)	0.403 (0.004)	0.112	0.021–0.585
**MSP9**	9.256 (0.002)	0.426 (0.002)	0.125	0.030–0.529

See Methods section for calculation of values. CI is confidence interval and OR, odds ratio.

**Table 3:** Adjusted effects of categorized variables which predict a lower risk for symptoms in a multivariate model

**Variables**	**Log rank test**	**Odds ratio**	**Confidence interval 95% for OR**
**RI EBA175**	X^2^ (2) = 14.660		
**3° Tercile x 1° Tercile**	*p* < 0.001	8.000	1.686–37.951
**RI EBA175**	X^2^ (2) = 20,775 *p* < 0.000	5.757	1.119–29.627
**3° Tercile x 1° Tercile**			
**RI MSP5**		9.447	1.289–69.256
**3° Tercile x 2° Tercile**			

A significant result indicates that strong responders against the indicated antigens are more likely to remain symptom-free. The terciles inform which ranked groups of responses were compared. See the Methods section for calculation of values.

### IgG Subclasses slightly differ in patients with or without symptoms

**Figure 6:**
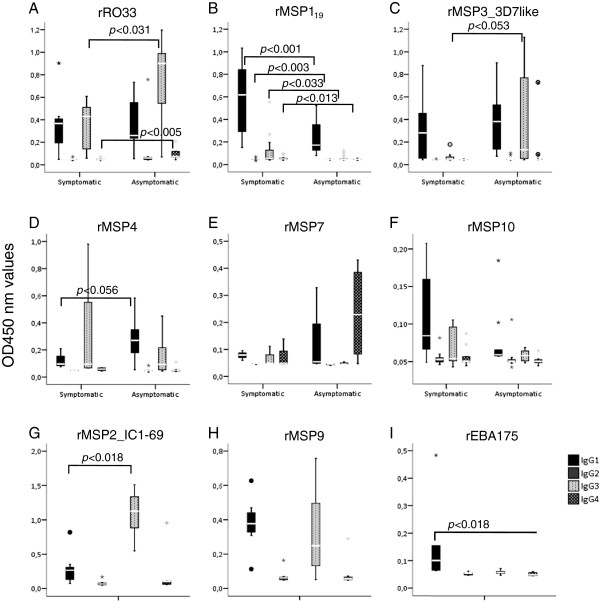
**IgG subclass distribution against strongly recognized recombinant antigens (RI > 10). ***p* values were calculated using Mann-Whitney's (U) test. A: IgG1 and IgG3 subclasses were predominant with differences among groups reactive to rRO33 (IgG3 asymptomatic (A) > IgG3 symptomatic (S)). The same was observed against rMSP4 with IgG1(A) > IgG1(S). IgG4 was present in reactive asymptomatic plasmas to rRO33 and in reactive symptomatic plasmas to rMSP1_19_. **B**: rMSP1_19_(IgG1(S) > IgG1(A), IgG3(S) > IgG3(A)). **C**: Against rMSP3-3D7-like, both groups presented IgG1 and a tendency to IgG3(A) > IgG3(S). **D**: A tendency to higher IgG1 in plasmas from asymptomatics was observed against rMSP4. **E/F**: No differences among groups to rMSP7 and rMSP10_3D7-like. IgG1, IgG3 and IgG4 were detected in symptomatic plasmas and IgG1 and IgG4 in asymptomatic plasmas with no differences among IgG subclasses for both groups. **G-I**: Only asymptomatic plasmas presented RI > 10 to rMSP2_IC1-69, rMSP9 and rEBA175. IgG3 were predominant in reactive asymptomatic plasmas to rMSP2_IC1-69 by Wilcoxon signed rank test and IgG1 were predominant in reactive asymptomatic plasmas to rMSP9, by Friedman’s test. No difference was observed between IgG1 and IgG3 against rEBA175 in reactive plasmas from asymptomatics.

Distinct IgG subclasses indicate the nature of the acquired immune response, pointing to the differential participation of components of cellular mediated immunity or inflammatory response regulation. In order to detect if the IgG subclass distribution was altered between the two clinical groups, plasmas that presented RIs > 10 against a given antigen were used to determine the IgG subclasses involved in the recognition of the respective antigen. In plasmas of symptomatic and asymptomatic patients, we observed differences in the IgG subclass profile in their response against the RO33 type of MSP1 block 2 and rMSP1_19_, but no significant differences (although borderline) were seen against rMSP3_3D7-like and rMSP4 (Figure 6). In both groups, the predominant IgG subclasses reactive against RO33 were IgG1 and IgG3. Plasmas from asymptomatic patients presented with higher quantities of IgG3 than plasmas from symptomatic patients (U = 11, *p* < 0.031) and the same was true for IgG4 (U = 6, *p* < 0.005). All subclasses of antibody against rMSP1_19_ were higher in plasmas from symptomatic patients: IgG1 (U = 43, *p* < 0.000), IgG2 (U = 75.5, *p* < 0.003), IgG3 (U = 100, *p* < 0.033) and IgG4 (U = 89.5, *p* < 0.013), but IgG1 was predominant in both groups. Plasmas from symptomatic infections presented with a predominant response of IgG1 against rMSP3_3D7-like while the predominant subclasses were IgG1 and IgG3 in asymptomatic infections. However, there was no difference in IgG1 among plasmas from both groups but a tendency to a higher quantity of IgG3 in plasmas from asymptomatic carriers (U = 25.5, *p* < 0.053). The predominant antibody subclasses to rMSP4 were also IgG1 and IgG3, but no differences between plasmas from both groups were observed. Nevertheless, a tendency towards higher quantities of IgG3 in plasmas from the asymptomatic patients was noted (U = 7, *p* < 0.056). No subclass differences were observed against the antigens rMSP7 and rMSP10_3D7-like, in the plasmas for which titers were measured. The predominant subclasses reactive against rMSP7 were IgG1, IgG3 and IgG4 while IgG1 and IgG3 were increased against rMSP10_3D7-like. All plasmas used to determine the subclasses reactive against antigens rMSP2, rMSP9 and rEBA175 belonged to the asymptomatic group, since only these presented RIs higher than 10. The predominant IgG subclass against MSP2 was IgG3 (Wilcoxon signed rank test, *p* < 0.018), while IgG1 was found against rMSP9 (Wilcoxon signed rank test, *p* < 0.033). No differences in the IgG subclasses were found against rEBA175 (Figure 6).

The duration of the humoral response, seen as the maintenance of antibody titers against target proteins, is at least partly expected to be important for the control of parasitemias. In order to address the longevity of the IgG response against determined targets, a subgroup of 10 asymptomatic and 3 symptomatic patients had their blood samples collected at 30 and 60 days after the first blood retrieval and treatment (these follow-up data are not included in Table 1). Due to the small number of plasmas, these were analyzed together. While the antibody quantities measured as reactivity indices against rMSP1 block 2 (RO33) and rMSP3_3D7-like decreased over time, the antibody levels against rMSP1 block 2 (MAD20 and K1) and rMSP2_FC27 did not change between T0 and T30, but decreased at T60. Antibodies against rMSP2_IC1-69, rMSP2_IC1-I4, rMSP6 and rMSP7 increased by T30 and then decreased by T60. No significant fluctuation of antibody levels was found against the remaining antigens (Table 4 and Additional file 2: Figure S5). These results underscore that the boosting of antibodies and the induction of memory is different from antigen to antigen.

**Table 4:** Different antigens elicit differently enduring IgG responses

**rAntigens**	***p*** **< (T0 > T30)**	***p*** **< (T30 > T60)**
MSP1bl2_RO33	0.028	0.015
MSP3_3D7-like	0.022	0.033
	***p*** **< (T0 < T30)**	***p*** **< (T30 > T60)**
MSP1bl2_MAD20	NS	0.028
MSP1bl2_K1	NS	0.043
MSP2_FC27	NS	0.018
MSP7	0.05	0.025
	***p*** **< (T0 < T30)**	***p*** **< (T30 > T60)**
MSP2_IC1-69	0.043	NS
MSP2_IC1-I4	0.026	NS
MSP6	0.011	NS
	***p*** **< (T0 > T30)**	***p*** **< (T30 > T60)**
MSP1_19_	NS	NS
MSP4	NS	NS
MSP5	NS	NS
MSP9	NS	NS
MSP10 (3 alleles)	NS	NS
AMA1(2 peptides)	NS	NS
EBA140	NS	NS
EBA175	NS	NS
EBA181	NS	NS

The significance of differences in the longevity of the humoral response in 13 serial samples (at 0, 30 and 60 days) is shown. For example, T0 > T30 means that the average of RI values decreased at time point 30. *NS* means statistically not different (Wilcoxon signed rank test). See also Additional file 2: Figure S5 for reactivity indices at time points 0, 30 and 60.

## Discussion

Immunity to blood stage malaria is seen as a process that takes several years of continued exposure to infection and reasons for this delay to a protective response may lie in the suppression of an effective T cell response [36,37] and the great diversity of antigens exposed to the host immune system. There is striking evidence that the immune response mediated by antibodies against either the infected red blood cell surface or the merozoite is of prime importance in the observed partial protection of individuals [38]. It appears that at least two types of a humoral response against blood stage malaria may lead to clinical protection. On one hand, the vaccine-induced production of function-inhibiting antibodies against otherwise rarely recognized antigens, such as the PfRh5 antigen [39], may lead to protection. On the other hand, a significant degree of protection mediated by antibodies is induced through years of exposure to natural infection with multiple parasite lines and antigens. In our study we tried to elucidate which antigen or combination of antigens was recognized in order to provide partial protection (asymptomatic status). In contrast to the high throughput assay performed by Crompton and colleagues [40], we analyzed antigens which are expected to contribute to immunity since they are exposed on the merozoite’s surface.

In the studied population, the individuals who suffered from symptomatic infections showed almost identical epidemiologic features when compared to the asymptomatic individuals. The variable TSLE (time since the last falciparum symptomatic episode, Tables 1, 2 and 3) pointed to the magnitude of the clinical immunity developed by asymptomatic patients. A significant number of individuals were not only asymptomatic at the onset of the study but indeed had been symptom-free for up to 30 years regardless of continued exposure to the parasite. The symptomatic group consisted of individuals with relatively mild malaria symptoms and low parasitemias and no single case of truly severe malaria was observed in the area. Severe malaria itself is a rare event in the Brazilian Amazon and the reasons for this may be rapid access to adequate individual antimalarial treatment, restricted virulence antigen repertoires in circulating strains [27], low transmission rates with *Anopheles darlingi*[24,41] being the principal vector, and partly due to successful efforts to control malaria in the Amazon region during the last 20 years [22]. Given the similar history of exposure to *P. falciparum* in the herein tested symptomatic and asymptomatic patients groups, it can be assumed that the former group is in the process of acquiring clinical immunity to malaria and will ultimately become part of the asymptomatics group. The difference between the clinical status of each group lies in the step at which their members are at in the process to clinical immunity. Importantly, the sample number of 51 in our experiments is low compared to studies in other endemic settings such as found in Africa or South East Asia. Therefore, it would be important to confirm our results in future studies using larger sample sizes. However, in the current situation of low transmission and decreasing incidence of *Plasmodium falciparum*, it is logistically difficult to achieve this in most areas of the Amazon.

When amplifying merozoite-protein related genes, many negative results in the amplification step were obtained and these occurred mostly in samples from asymptomatic infections. There are two main possibilities which may account for this observation: Firstly, the low parasitemia in asymptomatic infections and consequently the small quantity of recovered plasmodial genomic DNA may have been insufficient to permit the amplification of determined genes. Secondly, it is possible that distinct polymorphic sequences - different from the 3D7 genome – were present at the primer binding sites in isolates especially from asymptomatic infections. However, even when analyzing the alleles of the highly polymorphic block 2 of MSP1, the quantity of different sequences found were much smaller than previously described in high transmission malaria areas in Africa [34], but comparable to previous studies in the Amazon [42]. The rather small number of different sequence types in the circulating field isolates supports previously published data regarding the diversity of the repertoire of *var* variant gene DBL-α sequences in circulating isolates from the same area [27].

In *P. falciparum* donor samples from the same area, a differentiation of parasite haplotypes associated to asymptomatic or symptomatic donors was observed [43]. When we clustered the obtained sequences for each analyzed gene according to the criterion “symptomatic” or “asymptomatic”, we detected a significant accumulation of isolates with MSP1, block 2 sequence type K1 in parasites from asymptomatic patients while symptomatic patients showed a higher proportion of parasites with the RO33 type sequence. The reason for this is unclear and, to our knowledge, has never been described in other reports. All other sequences from antigens included in the study showed high similarity to sequences from the 3D7 genome, reinforcing a limited diversity of relevant protein sequences in the Amazon.

We then asked if there were antigens (or groups of antigens) which were recognized differentially by asymptomatic or symptomatic carriers. One has to take into account that the response in symptomatic patients may reveal higher antibody titers against certain antigens. This may lead to the probably erroneous interpretation that the presence of antibodies is detrimental for the health condition of the respective carrier. Also, the quality of antigens used may not permit exact conclusions about the functionality of the detected antibodies. Many of the antigens contain conformational epitopes in their native full-length form and these were probably not reproduced in our recombinant antigens, likely restricting the assay to the detection of antibodies against linear epitopes. At least rMSP1_19_ and perhaps rEBA175, however, contained conformational epitopes which could be recognized by antibodies. This was seen by the heat-mediated denaturation of these proteins leading to a decrease in their recognition (Additional file 2: Figure S3).

Testing of the leading erythrocyte stage vaccine candidates MSP1_19_, MSP2, MSP3 and AMA1 [20] has revealed interesting features in the development of clinical immunity to falciparum malaria in these low endemic areas. The first falciparum episodes normally are sufficient to generate IgG antibodies to MSP1_19_. Intriguingly, some asymptomatic patients exhibited no antibodies to this antigen suggesting that antibodies against solely this domain are not critical for an asymptomatic outcome, regardless of its importance in the invasion process and parasite survival [44]. Nevertheless, this antigen was largely recognized in almost all patients in both groups and the antibody response was maintained over time. The IC1 allele of MSP2 was largely recognized in both groups and the same was true for the rarely occurring FC27 allele, although to a lesser extent but still at a higher rate than may be expected from the genotyping of circulating strains. The presence of antiMSP2_FC27 either corroborates the presence of shared epitopes between polymorphic sequences or a previous, more abundant presence of the FC27 allele. A similar result was observed in the study by Jordan and colleagues regarding the occurrence of antibodies against determined allelic forms of MSP3 [45] and this seems to be a feature for areas with low transmission conditions such as those found in the Western Amazon. It is known that MSP1 and MSP6 genotypes fluctuate in the Amazon area over time [42,46] and this is probably true for MSP2 genotypes. No differences regarding the intensity of the immune response of both clinical groups were observed, despite the higher RI values to MSP2 antigens found in the asymptomatic group. To our knowledge, no study aiming to associate the anti-MSP2 response and protection has previously been done in the Amazon. In a study analyzing immune responses and protection from clinical malaria in the Gambia, a combined higher immune response against MSP2 alleles and AMA1 was associated to protection [47]. Polley and colleagues also found a strong correlation between antibodies to MSP2 and protection [48]. IgG3 was the predominant subclass for IC1 and also for rMSP1_19_ among asymptomatic plasmas and may be associated to the asymptomatic profile, interacting with phagocytes and favoring ADCC mechanisms, such as the respiratory burst by neutrophils [49]. This is in concordance with the data from other groups in settings of higher endemicity (e.g. [50] and papers cited therein).

The polypeptide MSP3_3D7-like, the major MSP3 allele found among the Rondonian isolates, was broadly recognized by plasmas from asymptomatic and symptomatic patients, however, with no differences that were statistically significant. In a recent study monitoring indigenous populations from the Venezuelan Amazon, smaller numbers of individuals with antibodies against MSP3 (11.2%) compared to our study (70%) were found, which is possibly due the fact that the study groups in that work contained only a few parasite carriers [51]. Another study in the Peruvian Amazon also detected a predominance of one distinct MSP3 genotype and a similar recognition frequency of the MSP3 HB3 allelic form (very similar to the 3D7 sequence) as found herein [45]. In that study, the responses to the N-terminal and the conserved C-terminal region were measured separately and the authors detected a higher recognition of the polymorphic N-terminal portion of MSP3 compared to the C-terminus. Our study used a peptide including the variant N- and the conserved C-terminal region of the MSP3_3D7 antigen, making us unable to dissect the response either against the polymorphic N or the conserved C-terminus as done in the study of Jordan and colleagues. Notably, the same group also observed the occurrence of MSP3 genotypes similar to those found in our study [52].

The interaction of antiMSP3 specific immunoglobulins with phagocytes and their role in ADCI mechanisms has been described previously [53] and the higher portion of IgG3 in asymptomatic individuals compared to symptomatic persons may underscore their importance in disease outcome. However, the antibody response to this important vaccine candidate seemed short-lived as shown by a fast decrease of the response in plasmas collected 30 or 60 days after the treatment. Perhaps for this reason, the IgG3 subclass in some asymptomatic plasma became predominant. These residual anti-MSP3 IgG3 antibodies may contribute to the maintenance of the asymptomatic profile, pointing to its importance in the acquisition of clinical immunity. In a study conducted in Senegal, the presence of IgG3 was significantly associated with protection [11]. Importantly, a MSP3 based vaccine proved efficient in a phase 1b vaccine trial [21].

The longer AMA1 peptide (rAMA1_B = M1 and M2 domains) showed a higher frequency of recognition than the shorter (rAMA1_A) corresponding to the M1 domain and the region between M1 and M2 domains. Analysis of AMA1 recognition by plasmas from Papua New Guinea showed that immune epitopes present in the M2 and M3 domains depend on the conformational structure of the protein for antibody interaction. The cysteine-rich M1 and M2 domains and their loop structures [54] contain conformational epitopes and are generally not well reconstituted in a prokaryotic expression system. The relatively weak recognition of the two peptides expressed (IR < 5 for AMA1_A and IR < 10 for AMA1_B) could be a consequence of the probable linear structure of the recombinant peptides used in our trials. When monitoring antibody titers in subsequent samples after treatment, no difference in recognition by plasmas from asymptomatic and symptomatic individuals was observed. Interestingly, the number of reactive plasmas among those collected 30 and 60 days after treatment increased over time. It is known that there is an extensive polymorphism in the AMA1 peptide which is critical for immune escape [55]. Of note, the expressed peptides also contain this region but in our isolates there was only one AMA1-allele in circulation, in contrast to what was found in other endemic regions [56]. Another tested antigen that probably suffers from incorrect folding during prokaryotic expression is MAEBL. Although we found only two different sequences, we noticed that these showed several amino acid changes present in their M1 and M2 domains.

The immune response developed to MSP1 block 2 has been associated with protection by some groups [57,58], but not by others [34,59,60]. *Aotus* and *Saimiri* monkeys immunized with native or recombinant MSP1 were protected when challenged with homologous or heterologous *P. falciparum* strains [57,61]. These results indicated that the C-terminal as well as the N-terminal regions of MSP1 can be associated to protection. This also raises the possibility of immune crossreaction among the allele families. Here, among the 24 recombinant peptides tested in ELISA assays, the RO33 type-sequence of MSP1 block 2 generated the highest RI values (RI > 100), but these were not restricted to one clinical group. Regardless of the decrease in RI values observed in plasmas from asymptomatic patients collected 30 and 60 days after treatment, the subclasses identified in plasmas collected before treatment showed a predominance of cytophilic antibodies IgG1 and IgG3 in both clinical groups, but asymptomatics exhibited higher IgG3 quantities than symptomatics. Again, it is possible that better protection is due to the interaction of IgG3 and Fc receptors on the phagocytes’ surface, triggering mechanisms of ADCI described previously for this antigen [62]. Interestingly, an IgG4 response was observed against the RO33 type-sequence of MSP1 block 2, and this was stronger in asymptomatic than in symptomatic patients and extended to MSP6 and MSP7 which form a complex together with MSP1. When measured, the antibody response to MSP7 decreased over time, but in the plasmas collected before treatment, IgG1 and IgG4 were the predominant antigens with no differences among the clinical groups. Few studies have addressed the humoral response against MSP6 and 7 in populations, and none have been done in Brazil. The recognition frequency of MSP6 and 7 seen in our study group was significantly lower than in malaria patients tested in India [63], perhaps due to different exposure of patients or conformational issues of the proteins used. In a recent study from the Peruvian Amazon, a comparable recognition of MSP6 (C-terminal fragment as used herein) was found [64]. MSP6 is found in two allelic forms in *P. falciparum*, and we found a predominance of the 3D7 sequence, in accordance with results by Neal and colleagues who analyzed isolates from the Iquitos region [46].

The only antigens for which the intensity of the antibody response revealed differences in the recognition between symptomatic and asymptomatic plasmas were rMSP4, rMSP5, rMSP9 and rEBA175. The first two have a conserved sequence among different strains of *P. falciparum* but unknown functions [65]. The acid-basic repeat antigen (ABRA or MSP9) has been linked to the proteolytic processing of band 3 protein [66], the putative erythrocyte receptor for MSP1_19_[67]. EBA175 is the most important antigen implicated in the invasion of erythrocyte by the sialic acid dependent invasion pathway [68]. rMSP4 and rEBA175 were widely recognized by plasmas from both clinical groups but revealed a stronger response in plasmas from asymptomatic patients. Although rMSP5 was readily amplified in parasites from asymptomatic and symptomatic infections, the antibody response was restricted to the asymptomatic group. Independently from the low RI observed in the ELISA assays using rMSP5, the presence of antibodies to this antigen and its intensity of recognition predicted significantly higher chances for the absence of symptoms and the development of the asymptomatic profile, respectively. Notably, MSP5 is not essential for parasite survival *in vitro*[69]. The antibody response against MSP4 and MSP5 has never previously been tested in Amazonian settings, and the overall recognition of MSP5 was comparable to a recent study done in Papua New Guinea (PNG), although we found much less recognition in symptomatic individuals compared to the study in PNG [70]. Perhaps for this reason, that study also did not reveal a differential intensity of recognition between asymptomatic and symptomatic individuals. A result similar to what was found for rMSP5 was observed for rEBA175 while the magnitude of antibody response to rMSP9 only predicted higher chances for the absence of symptoms. High levels of antibodies against EBA175 were also shown to be protective against symptomatic malaria in children in PNG [71], while no comparable study has previously been done in the Amazon. Also, and to our best knowledge, no study has been conducted which measured PfMSP9-specific antibodies in natural infections. The correlation between the antibody response against it and the asymptomatic status surely makes this molecule an interesting target.

Our analysis revealed that the main potential vaccine antigens MSP1_19_, MSP2, MSP3 and AMA-1 were recognized equally in number and intensity by both groups of patients. Despite all concerns regarding the quality of the expressed antigens and/or the restricted antigen repertoire in the Brazilian Amazon, it appears that the recognition of some of these antigens is dispensable for the development of an asymptomatic profile or is another indication that we are observing very similar groups in relation to the clinical immunity to falciparum malaria. On the other hand, what is the real importance of developing antibodies against MSP4, MSP5, MSP9 and EBA-175, which were the only antigens tested in our study recognized more by asymptomatic patients? Is the recognition of only one single antigen as effective as the recognition of all four antigens?

To answer these questions, we performed the cross tab and the logistic regression analysis. The multivariate model was employed in the logistic regression to measure the independent contribution of each antigen to the asymptomatic profile and this was achieved by the estimation of the odds for this outcome. The only antigens independently associated with the asymptomatic profile were MSP5, MSP9 and EBA-175. This means that the presence of antibodies against each of them is necessary to generate or maintain the asymptomatic profile. Due to this, a potential vaccine based on single merozoite antigen would not likely induce the desirable protective status, while a vaccine based on multiple merozoite antigens may be better. Accordingly, the multiple target approach proved more effective in *in vitro* approaches to measure the quality of vaccines (e.g. [72]). Considering that the several existent parasite invasion routes are redundant, the development of the asymptomatic condition will require the recognition of multiple antigens involved in each of these routes. Two antigens, implicated in two different invasion routes (MSP9 and EBA-175) were independently classified as important for the development of the asymptomatic status, while the third protein, MSP5, has still no assigned function and is also non-essential [69]. It is important to note that the univariate model of logistic regression defined odds ratios associated MSP3_3D7 and MSP4 to the asymptomatic status (data not shown). However, the multivariate model which permits the visualization of the independent variables for a specific outcome did not confirm this result. Further tests, perhaps with higher sample numbers, are necessary to elucidate this issue especially when considering that MSP3 is a vaccine candidate already used in clinical field trials [21].

## Conclusions

The central question in the discovery of relevant vaccine targets is how one can differentiate a protective antibody response from a response generated through the ongoing exposure but which is still unable to provide clinical protection. When divided into the two clinical groups representing immune (asymptomatic – “protected”) and semi-immune (symptomatic – “susceptible”) persons almost no differences among the exposure variables to *P. falciparum* parasites were noted and also an absence of differences in the immune response generated to the majority of merozoite antigens was observed. Of these, some are known to be involved in the erythrocyte invasion such as MSP1, AMA1 and EBA-175, while the others are probably involved directly or indirectly in the same process. Our results point to the hypothesis that the acquisition of clinical immunity based on the humoral response is probably a cumulative step- by-step process of expansion and refinement of an antibody repertoire that reacts to a certain number of merozoite antigens. One of the first recognized antigens is apparently MSP1_19_ because of the ample reaction of plasmas from both clinical groups. The maintenance of an antibody response to the majority of merozoite antigens indicates the development and expansion of specific long-lived plasma cells in this cumulative immune response [73]. The maintenance of exposure to falciparum parasites then seems to enable the recognition of other ligands important for invasion, such as EBA175, to which an intense antibody response was restricted to the asymptomatic group. Probably, the development of clinical immunity requires the recognition of a greater number of antigens that exert the same function in redundant invasion pathways. Additionally, the recognition of antigens that interact with important antigens that are involved in the invasion process, such as MSP9, may be important. The antibody response to the other polymorphic antigens was similar among the two clinical groups, with no differences in the frequency or intensity of the humoral immune response. In regard to the conserved antigens MSP4 and MSP5, asymptomatic plasmas exhibited differential recognition patterns and antibodies to MSP5 were associated with protection from clinical symptoms and the development of the asymptomatic profile. While MSP4 is considered essential to parasite survival, MSP5 is not [69]. Our data seem to indicate that merozoite antigens may predominantly induce cytophilic antibodies that then probably interact with PMBC, triggering ADCI mechanisms. In accordance to what was shown by previous studies, the development of an antibody response associated to protection requires the recognition of multiple antigens that act in redundant invasion pathways. The antibody response to essential conserved antigens, not under immune pressure, is perhaps the last step for the development of a protective antibody immune response.

## Competing interests

The authors declare that they have no competing interests.

## Authors’ contributions

MMM and GW conceived the study, MMM, WLF and GW performed the experiments, MMM, RCDM, THK and LHPdS collected samples, recorded and analyzed patient information and provided infrastructure for the processing of samples, MMM and GW wrote the manuscript. All authors read and approved the final manuscript.

## Pre-publication history

The pre-publication history for this paper can be accessed here:

http://www.biomedcentral.com/1471-2334/13/608/prepub

## Supplementary Material

Additional file 1: Table S1 Genotype diversity of amplified fragments of merozoite genes (columns) per Pf field isolates (lines) in dashed (1 = MSP1 block 2) and color codes (other genes). White color = unsuccessful amplifications and black color = 3D7-type sequences. 1 to 10 = MSP1 block 2 to MSP10, 11A = AMA1_A, 11B = AMA1_B, 12 = MAEBL, 13 = EBA140, 14 = EBA175 and 15 = EBA181. I1, I2, I3, I4 = isolates previously collected in the same settings. AFI = Africa Field Isolate (Gabon). The dominant sequence of each protein was used for expression and the sequences of the used sequences can be found under the following GenBank accessions: RO33-JX315617, MAD20A-JX412318, MAD20B-JX412319, MAD20C-JX412320, MAD20D-JX412321, MAD20FCR3-JX412322, K1A-JX416338, K1B-JX416339, K1C-JX416340, K1AFI-JX416341, MSP2FC27-JX424324, MSP2_IC1-69-JX424323, MSP2_IC1_I4(3D7-like)-JX469122, MSP3K1-like-JX469137, MSP4-JX469123, MSP5-JX469124, MSP6-JX469125, MSP7-JX469126, MSP9-JX469127, MSP10_17-JX469128, MSP10_I3-JX469129, MSP10_3D7-like-JX469130, AMA1-JX469131, EBA140-JX469132, EBA175-JX469133, EBA181-JX469134, MAEBL_I4-JX469135, MAEBL_3D7-like-JX469136. The different MSP1 block 2 types are shown below. Repeat motifs in K1 and MAD20 sequences and the RO33 peptide sequence from *P. falciparum* field isolate gDNAs. The references where the sequences were found previously are indicated in the right column. **Table S2.** Primer pairs to amplify specific fragments of merozoite expressed genes. The properties of the recombinant peptides are shown: C, conserved; P, polymorphic; ND - no specific property described for the full length protein. Inserted restriction sites are shown in **bold** letters. F, forward and R, reverse primer. Click here for file

Additional file 2: Figure S1 Primer localization and amplified sequences in coding regions of genes encoding tested antigens. Gene models were extracted from PlasmoDB.org v5.5. **Figure S2.** Coomassie stained, denaturing 8% SDS-PAGE of GST-fused proteins used in ELISAs: **A**: 1: GST, 2–4, MSP1 block 2 alleles RO33, MAD20 and K1, 5–7: MSP2 alleles FC27, IC1_69, IC1_3D7like, 8–9 MSP3 alleles K1 and 3D7, 10: MSP4, 11: MSP5, 12: MSP6, 13, MSP7, 14: MSP8 (not tested in ELISAs), 15: MSP9. **B**: 1: GST, 2–4: MSP10, 17, 369 and 3D7, respectively, 5: AMA1_A 6: AMA1_B, 7: EBA140, 8: EBA175, 9: EBA181, 10–11: MAEBL type I4 and 3D7. **Figure S3.** Recombinant GST-fused antigens partially possess conformational epitopes which are destroyed upon heat denaturation. ELISAs were performed as before using the indicated antigens either heated for 5 min at 95°C or not. The upper graph shows the percentage of recognition of heated antigens from four strongly and two weakly reacting plasmas from asymptomatic individuals (corresponding to plasmas in lanes 4, 5, 6, 11, 12, 13 in Table 1). The lower graph shows the OD450nm values for unheated antigens. **Figure S4.** Reactivity of plasmas from infections with determined MSP1-block2 alleles against antigen variants. No statistical difference was observed in the response of plasmas from carriers of the given MSP1 block 2 genotypes and their reaction against MSP1 block 2 antigens. See Additional file 1: Table S1 for details of the genotypes. **Figure S5.** Reactivity indices (y-axis) of sera against antigens in the follow-up analysis (day 0, 30 and 60), shown are median values (horizontal line), 25-75% percentiles (boxes) with their deviations and outliers (asterisks). Only values of antigens which were recognized statistically different between at least two time points are shown (see Table 4 for details). Click here for file
