# Supplementary material for: Natural antibody response to Plasmodium falciparum merozoite antigens MSP5, MSP9 and EBA175 is associated to clinical protection in the Brazilian Amazon
Source: BMC Infect Dis. 2013 Dec 28;13:608. doi: 10.1186/1471-2334-13-608 (PMC3880555; doi:10.1186/1471-2334-13-608)
Supplement: Additional file 1: Table S1 — Genotype diversity of amplified fragments of merozoite genes (columns) per Pf field isolates (lines) in dashed (1 = MSP1 block 2) and color codes (other genes). White color = unsuccessful amplifications and black color = 3D7-type sequences. 1 to 10 = MSP1 block 2 to MSP10, 11A = AMA1_A, 11B = AMA1_B, 12 = MAEBL, 13 = EBA140, 14 = EBA175 and 15 = EBA181. I1, I2, I3, I4 = isolates previously collected in the same settings. AFI = Africa Field Isolate (Gabon). The dominant sequence of each protein was used for expression and the sequences of the used sequences can be found under the following GenBank accessions: RO33-JX315617, MAD20A-JX412318, MAD20B-JX412319, MAD20C-JX412320, MAD20D-JX412321, MAD20FCR3-JX412322, K1A-JX416338, K1B-JX416339, K1C-JX416340, K1AFI-JX416341, MSP2FC27-JX424324, MSP2_IC1-69-JX424323, MSP2_IC1_I4(3D7-like)-JX469122, MSP3K1-like-JX469137, MSP4-JX469123, MSP5-JX469124, MSP6-JX469125, MSP7-JX469126, MSP9-JX469127, MSP10_17-JX469128, MSP10_I3-JX469129, MSP10_3D7-like-JX469130, AMA1-JX469131, EBA140-JX469132, EBA175-JX469133, EBA181-JX469134, MAEBL_I4-JX469135, MAEBL_3D7-like-JX469136. The different MSP1 block 2 types are shown below. Repeat motifs in K1 and MAD20 sequences and the RO33 peptide sequence from P. falciparum field isolate gDNAs. The references where the sequences were found previously are indicated in the right column. Table S2. Primer pairs to amplify specific fragments of merozoite expressed genes. The properties of the recombinant peptides are shown: C, conserved; P, polymorphic; ND - no specific property described for the full length protein. Inserted restriction sites are shown in bold letters. F, forward and R, reverse primer. [file 1471-2334-13-608-S1.docx]

**Supplementary Table 1:** Genotype diversity of amplified fragments of merozoite genes (columns) per Pf field isolates (lines) in dashed (1=MSP1 block 2) and color codes (other genes). White color = unsuccessful amplifications and black color=3D7-type sequences. 1 to 10 = MSP1 block 2 to MSP10, 11A = AMA1_A, 11B = AMA1_B, 12 = MAEBL, 13 = EBA140, 14 = EBA175 and 15 = EBA181. I1, I2, I3, I4=isolates previously collected in the same settings. AFI=Africa Field Isolate (Gabon). The dominant sequence of each protein was used for expression and the sequences of the used sequences can be found under the following GenBank accessions: RO33-JX315617, MAD20A-JX412318, MAD20B-JX412319, MAD20C-JX412320, MAD20D-JX412321, MAD20FCR3-JX412322, K1A-JX416338, K1B-JX416339, K1C-JX416340, K1AFI-JX416341, MSP2FC27-JX424324, MSP2_IC1-69-JX424323, MSP2_IC1_I4(3D7-like)-JX469122, MSP3K1-like-JX469137, MSP4-JX469123, MSP5-JX469124, MSP6-JX469125, MSP7-JX469126, MSP9-JX469127, MSP10_17-JX469128, MSP10_I3-JX469129, MSP10_3D7-like-JX469130, AMA1-JX469131, EBA140-JX469132, EBA175-JX469133, EBA181-JX469134, MAEBL_I4-JX469135, MAEBL_3D7-like-JX469136. The different MSP1 block 2 types are shown below.

| ***Pf* gDNAs** | **MSP1block2** | | | | **MSP2** | | **MSP3** | | **MSP4** | **MSP5** | **MSP7** | **MSP9** | **MSP10** | **AMA1_A** | **AMA1_B** | **MAEBL** | **EBA140** | **EBA175** | **EBA181** |
| --- | --- | --- | --- | --- | --- | --- | --- | --- | --- | --- | --- | --- | --- | --- | --- | --- | --- | --- | --- |
| **Field**  **Isolates**  **Collected**  **From**  **Asymptomatic**  **Patients** |  | | | |  | |  | |  |  |  |  |  |  |  |  |  |  |  |
|  |  | | | |  | |  | |  |  |  |  |  |  |  |  |  |  |  |
|  |  | | | |  | |  | |  |  |  |  |  |  |  |  |  |  |  |
|  |  | | | |  | |  | |  |  |  |  |  |  |  |  |  |  |  |
|  |  | | | |  | |  | |  |  |  |  |  |  |  |  |  |  |  |
|  |  | | | |  | |  | |  |  |  |  |  |  |  |  |  |  |  |
|  |  | |  | |  | |  | |  |  |  |  |  |  |  |  |  |  |  |
|  |  | | | |  | |  | |  |  |  |  |  |  |  |  |  |  |  |
|  |  | | | |  | |  | |  |  |  |  |  |  |  |  |  |  |  |
|  |  | |  | |  | |  | |  |  |  |  |  |  |  |  |  |  |  |
|  |  | | | |  | |  | |  |  |  |  |  |  |  |  |  |  |  |
|  |  | | | |  | |  | |  |  |  |  |  |  |  |  |  |  |  |
|  |  | | | |  | |  | |  |  |  |  |  |  |  |  |  |  |  |
|  |  | | | |  | |  | |  |  |  |  |  |  |  |  |  |  |  |
|  |  | | | |  | |  | |  |  |  |  |  |  |  |  |  |  |  |
|  |  | | | |  | |  | |  |  |  |  |  |  |  |  |  |  |  |
|  |  | |  | |  | |  | |  |  |  |  |  |  |  |  |  |  |  |
|  |  | | | |  | |  | |  |  |  |  |  |  |  |  |  |  |  |
|  |  | | | |  | |  | |  |  |  |  |  |  |  |  |  |  |  |
|  |  | |  | |  | |  | |  |  |  |  |  |  |  |  |  |  |  |
|  |  | | | |  | |  | |  |  |  |  |  |  |  |  |  |  |  |
|  |  | | | |  | |  | |  |  |  |  |  |  |  |  |  |  |  |
|  |  |  | |  |  | |  | |  |  |  |  |  |  |  |  |  |  |  |
|  |  | | | |  | |  | |  |  |  |  |  |  |  |  |  |  |  |
|  |  | | | |  | |  | |  |  |  |  |  |  |  |  |  |  |  |
|  | | | | | | | | | | | | | | | | | | | |
| **Field**  **Isolates**  **Collected**  **From**  **Symptomatic**  **Patients** |  | | | |  | |  | |  |  |  |  |  |  |  |  |  |  |  |
|  |  | |  | |  | |  | |  |  |  |  |  |  |  |  |  |  |  |
|  |  |  | |  |  | |  | |  |  |  |  |  |  |  |  |  |  |  |
|  |  | |  | |  | |  | |  |  |  |  |  |  |  |  |  |  |  |
|  |  | | | |  | |  | |  |  |  |  |  |  |  |  |  |  |  |
|  |  | | | |  | |  | |  |  |  |  |  |  |  |  |  |  |  |
|  |  | | | |  | |  | |  |  |  |  |  |  |  |  |  |  |  |
|  |  | | | |  | |  | |  |  |  |  |  |  |  |  |  |  |  |
|  |  | | | |  | |  | |  |  |  |  |  |  |  |  |  |  |  |
|  |  | | | |  | |  | |  |  |  |  |  |  |  |  |  |  |  |
|  |  | | | |  | |  | |  |  |  |  |  |  |  |  |  |  |  |
|  |  | | | |  |  |  | |  |  |  |  |  |  |  |  |  |  |  |
|  |  | | | |  | |  | |  |  |  |  |  |  |  |  |  |  |  |
|  |  | | | |  | |  | |  |  |  |  |  |  |  |  |  |  |  |
|  |  | | | |  | |  | |  |  |  |  |  |  |  |  |  |  |  |
|  |  | |  | |  | |  | |  |  |  |  |  |  |  |  |  |  |  |
|  |  | | | |  | |  | |  |  |  |  |  |  |  |  |  |  |  |
|  |  | | | |  | |  | |  |  |  |  |  |  |  |  |  |  |  |
|  |  | | | |  | |  | |  |  |  |  |  |  |  |  |  |  |  |
|  |  | | | |  | |  | |  |  |  |  |  |  |  |  |  |  |  |
|  |  | |  | |  | |  | |  |  |  |  |  |  |  |  |  |  |  |
|  |  | | | |  | |  | |  |  |  |  |  |  |  |  |  |  |  |
|  |  | | | |  | |  | |  |  |  |  |  |  |  |  |  |  |  |
|  |  | | | |  | |  | |  |  |  |  |  |  |  |  |  |  |  |
|  |  | | | |  | |  | |  |  |  |  |  |  |  |  |  |  |  |
|  |  | | | |  | |  | |  |  |  |  |  |  |  |  |  |  |  |
|  |  | | | |  | |  | |  |  |  |  |  |  |  |  |  |  |  |
|  | | | | | | | | | | | | | | | | | | | |
| **I1** |  | | | |  | |  | |  |  |  |  |  |  |  |  |  |  |  |
| **I2** |  | | | |  | |  | |  |  |  |  |  |  |  |  |  |  |  |
| **I3** |  | | | |  | |  | |  |  |  |  |  |  |  |  |  |  |  |
| **I4** |  | | | |  | |  | |  |  |  |  |  |  |  |  |  |  |  |
| **S20** |  | | | |  | |  |  |  |  |  |  |  |  |  |  |  |  |  |
| **AFI** |  | | | |  | |  | |  |  |  |  |  |  |  |  |  |  |  |
|  | | | | | | | | | | | | | | | | | | | |
| **FCR3** |  | | | |  | |  | |  |  |  |  |  |  |  |  |  |  |  |
| **3D7** |  | | | |  | |  | |  |  |  |  |  |  |  |  |  |  |  |

| **MSP1_block2 alleles** | **RO33** | **K1** | | | | | | | **MAD20** | | | | | |
| --- | --- | --- | --- | --- | --- | --- | --- | --- | --- | --- | --- | --- | --- | --- |
|  |  | **A** | **B** | **C** | | **AFI** | **3D7** | | **A** | **B** | | **C** | **D** | **FCR3** |
|  |  |  |  |  | |  |  | |  |  | |  |  |  |
| **MSP2 alleles** | **FC27** | | | | **IC1_69** | | | | | | **IC1_3D7 like** | | | |
| **MSP3 alleles** | **K1-like** | | | | | | | **3D7 like** | | | | | | |
| **MSP10** | **17** | | | | **I3** | | | | | | **3D7 like** | | | |
| **MAEBL** | **I4** | | | | | | | **3D7 like** | | | | | | |

**Supplementary Table 1 continued-** Repeat motifs in K1 and MAD20 sequences and the RO33 peptide sequence from *P. falciparum* field isolate gDNAs. The references where the sequences were found previously are indicated in the right column.

| Families | Codes | Nucleotide sequences | Repeat motifs | Names | Previously found in |
| --- | --- | --- | --- | --- | --- |
| K1 | 1  2  3  4 | AGT GGT ACA  AGT GGT CCA  AGT GCT CAA  AGT GGT GCA | 3 4 3 4 3 4 3 4 3 4 3 1 2 2 1  3 1 1 2 1  3 1 1 2 1 2 1 2 1 2 1 2 1  3 4 3 4 3 4 1  3 4 3 1 3 1 1 1 2 2 1 | K13D7 like  K1A  K1B  K1C  K1AFI | Africa [1]  Brazil [2,3]  Brazil [2,3] and Africa [1]  Africa [4]  Asia [5] |
|  | | | | | |
| MAD20 | 5  5  6  7  8  10 | TCA GGT GGT  TCA GGT GGC  TCA GTT GCT  TCA GTT ACT  TCA AAG GGT  TCA GGT AAT | 5 6 5 6 5 5 6 5 6 5 10  5 7 5 5 5 5 6 5 6 5 5 6 5 6 5 10  5 7 5 5 10  8 6 5 5 6 5 6 5 6 5 6 5 10  8 6 5 10 | MAD20-D  MAD20-A  MAD20-B  MAD20-FCR3  MAD20-C | Asia [6]  Brazil [3]  Asia [5]  Asia [5]  Asia [7] |
| RO33 | KPAGAVSTQSAKNPPGATVPSGTASTKGAIRSPGAANPSDDS | | | | |

**Supplementary Table 2** Primer pairs to amplify specific fragments of merozoite expressed genes. The properties of the recombinant peptides are shown: C, conserved; P, polymorphic; ND - no specific property described for the full length protein. Inserted restriction sites are shown in **bold** letters. F, forward and R, reverse primer.

| **Genes** | **Proteins** | **Expressed parts** | **Peptide characteristics** | **Immune epitopes** | **Primers pairs** |
| --- | --- | --- | --- | --- | --- |
| PFI1475w | **MSP1** | Block 2 (N–terminal) | P | B | **C1F_GGATCC**ctagaagctttagaagatgcag  **C3R_**acatatgattggttaaatcaaagag |
|  |  | C–terminal | EGF–like domains | B and T | **F_GGATCC**CCTAATACAATAATATCAAAATTAATTG  **R_ AGATCT**AGAGGAACTGCAGAAAATACC |
| PFB0300c | **MSP2** | Central | C and P | B and T | **F_GGATCC**AAGGTAATTAAAACATTGTC  **R_**GAGGGATGTTGCTGCTCCACAG |
| PF10_0345 | **MSP3_3D7** | Central | P | B | **F_GGATCC**ACTGGTAATGATTTTAGTGGTGG  **R_**AACGCCTCCTCCAAATTCCCAAC |
|  | **MSP3_K1** | Central | P | B | **F_GGATCC**ggaggttttactgcagatgatg  **R_**cgcctcctccaaattcccaacc |
| PFB0310c | **MSP4** | Exon 1 | C | B and T | **F_GGATCC**GGGGAAGAAAAACCAAATGTGG  **R_**CTACCTTTTTAGGGATAGCTTC |
| PFB0305 | **MSP5** | Exon 2 | C | ND | **F_GGATCC**CGAAAAAGGAGAAGGTGGTTTTAC  **R_**GGAGGTTCTTGACCTATTTCTGC |
| PF10_0346 | **MSP6** | C--terminal | C and P | B | **F_GGATCC**AGTAATAGTACTACAACTTCTC  **R_**CTACACTTTCTTCATCTATGTC |
| PF13_0197 | **MSP7** | C--terminal | C and P | ND | **F_GGATCC**ATGAATGATGACACATTCTTAGG  **R_**CACTTACAATATCCGTTTTGCC |
| PFE0120c | **MSP8** | C--terminal | P and domains EGF–like | T | **F_GGATCC**GAAGTAGATGCACTTTTGAAAAATG  **R_**AGGTAGACATCTACAGGTTTCTTC |
| PFL1385c | **MSP9** | N--terminal | P | B and T | **F_GGATCC**GCTACCTACTCTTTTGTTAATAC  **R_**TCATCCATACCAGATACAGTTTC |
| PFF0995c | **MSP10** | N--terminal | P | absent | **F_GGATCC**ACATCCCAGAAGAAAATTACATATG  **R_**CT**GAATTC**ATGATAGATGAATTTTC |
| MAL13P1.60 | **EBA140** | N--terminal | *Duffy-*like domains F1 and F2 | B (NKND) | **F_GGATCC**AGTAAAACACAAATGGAGGTTTTG  **R_**GTTTGTCTTCTGGGGGGTTCAC |
| MAL7P1.176 | **EBA175** | N--terminal | *Duffy-*like domains F1and F2 | B (NKND | **F_GGATCC**TGTGAGAAGGAATGTATTGATCC  **R_**GTTTCCAAGACATAATTCTTGCC |
| PFA0125c | **EBA181** | N--terminal | *Duffy-*like domain F2 | T | **F_GGATCC**TATGGTAGGAAAGGGGAATATTTG  CACCAAGCAGTAGGATTAAGTAC |
| PF11_0344 | **AMA1A** | N--terminal | PAN1 domain and IDR | T | **F_GGATCC**ACACCGGTAGCTACGGGAAATC  CAATTTCCATCGACCCATAATCCG |
|  | **AMA1B** | N--terminal | PAN1 and PAN2 domains | B and T | **F_GGATCC**AACACCGGTAGCTACGGGAAATC  AGCAGTAGTAGCAATGTATGATG |
| PF11_0486 | **MAEBL** | N--terminal | PAN2 domain | T | **F_GGATCC**AGAAGTCCATCTATAGGAG  GTCATATTCATTTTCCTTATA |

References for sequences from Supplementary table 1

1. Noranate N, Prugnolle F, Jouin H, Tall A, Marrama L, et al. (2009) Population diversity and antibody selective pressure to Plasmodium falciparum MSP1 block2 locus in an African malaria-endemic setting. BMC Microbiol 9: 219.

2. Scopel KK, Fontes CJ, Ferreira MU, Braga EM (2005) Plasmodium falciparum: IgG subclass antibody response to merozoite surface protein-1 among Amazonian gold miners, in relation to infection status and disease expression. Exp Parasitol 109: 124-134.

3. Ferreira MU, Ribeiro WL, Tonon AP, Kawamoto F, Rich SM (2003) Sequence diversity and evolution of the malaria vaccine candidate merozoite surface protein-1 (MSP-1) of Plasmodium falciparum. Gene 304: 65-75.

4. Juliano JJ, Porter K, Mwapasa V, Sem R, Rogers WO, et al. (2010) Exposing malaria in-host diversity and estimating population diversity by capture-recapture using massively parallel pyrosequencing. Proc Natl Acad Sci U S A 107: 20138-20143.

5. Tanabe K, Sakihama N, Walliker D, Babiker H, Abdel-Muhsin AM, et al. (2007) Allelic dimorphism-associated restriction of recombination in Plasmodium falciparum msp1. Gene 397: 153-160.

6. Jiang G, Daubenberger C, Huber W, Matile H, Tanner M, et al. (2000) Sequence diversity of the merozoite surface protein 1 of Plasmodium falciparum in clinical isolates from the Kilombero District, Tanzania. Acta Trop 74: 51-61.

7. Umar F, Dubey ML, Malla N, Mahajan RC (2006) Plasmodium falciparum: polymorphism in the MSP-1 gene in Indian isolates and predominance of certain alleles in cerebral malaria. Exp Parasitol 112: 139-143.
