# Supplementary material for: Natural antibody response to Plasmodium falciparum merozoite antigens MSP5, MSP9 and EBA175 is associated to clinical protection in the Brazilian Amazon
Source: BMC Infect Dis. 2013 Dec 28;13:608. doi: 10.1186/1471-2334-13-608 (PMC3880555; doi:10.1186/1471-2334-13-608)
Supplement: Additional file 2: Figure S1 — Primer localization and amplified sequences in coding regions of genes encoding tested antigens. Gene models were extracted from PlasmoDB.org v5.5. Figure S2. Coomassie stained, denaturing 8% SDS-PAGE of GST-fused proteins used in ELISAs: A: 1: GST, 2–4, MSP1 block 2 alleles RO33, MAD20 and K1, 5–7: MSP2 alleles FC27, IC1_69, IC1_3D7like, 8–9 MSP3 alleles K1 and 3D7, 10: MSP4, 11: MSP5, 12: MSP6, 13, MSP7, 14: MSP8 (not tested in ELISAs), 15: MSP9. B: 1: GST, 2–4: MSP10, 17, 369 and 3D7, respectively, 5: AMA1_A 6: AMA1_B, 7: EBA140, 8: EBA175, 9: EBA181, 10–11: MAEBL type I4 and 3D7. Figure S3. Recombinant GST-fused antigens partially possess conformational epitopes which are destroyed upon heat denaturation. ELISAs were performed as before using the indicated antigens either heated for 5 min at 95°C or not. The upper graph shows the percentage of recognition of heated antigens from four strongly and two weakly reacting plasmas from asymptomatic individuals (corresponding to plasmas in lanes 4, 5, 6, 11, 12, 13 in Table 1). The lower graph shows the OD450nm values for unheated antigens. Figure S4. Reactivity of plasmas from infections with determined MSP1-block2 alleles against antigen variants. No statistical difference was observed in the response of plasmas from carriers of the given MSP1 block 2 genotypes and their reaction against MSP1 block 2 antigens. See Additional file 1: Table S1 for details of the genotypes. Figure S5. Reactivity indices (y-axis) of sera against antigens in the follow-up analysis (day 0, 30 and 60), shown are median values (horizontal line), 25-75% percentiles (boxes) with their deviations and outliers (asterisks). Only values of antigens which were recognized statistically different between at least two time points are shown (see Table 4 for details). [file 1471-2334-13-608-S2.pptx]

## Slide 1
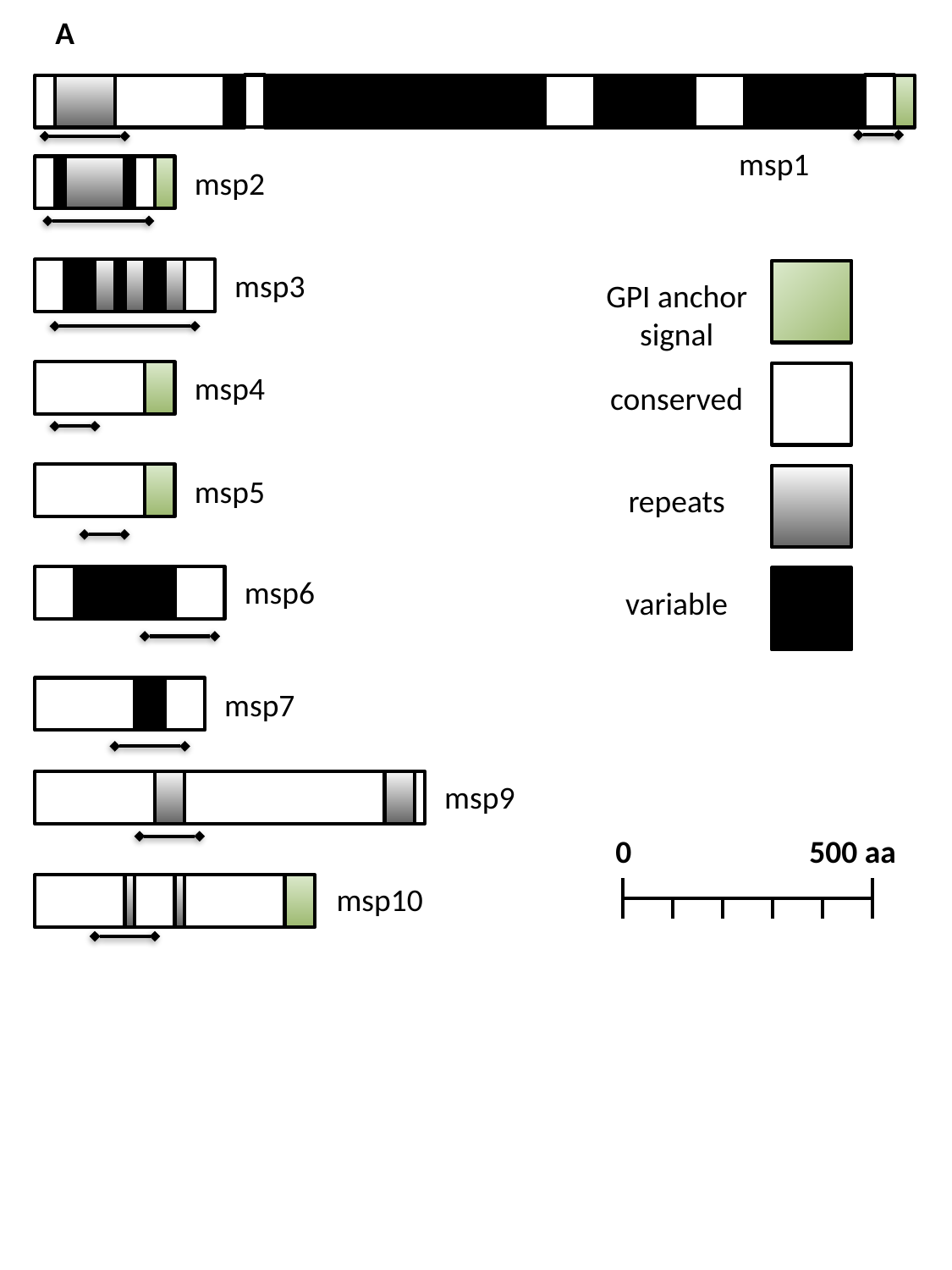

A
msp1
msp2
msp3
GPI anchor signal
msp4
conserved
msp5
repeats
msp6
variable
msp7
msp9
0
500 aa
msp10

## Slide 2
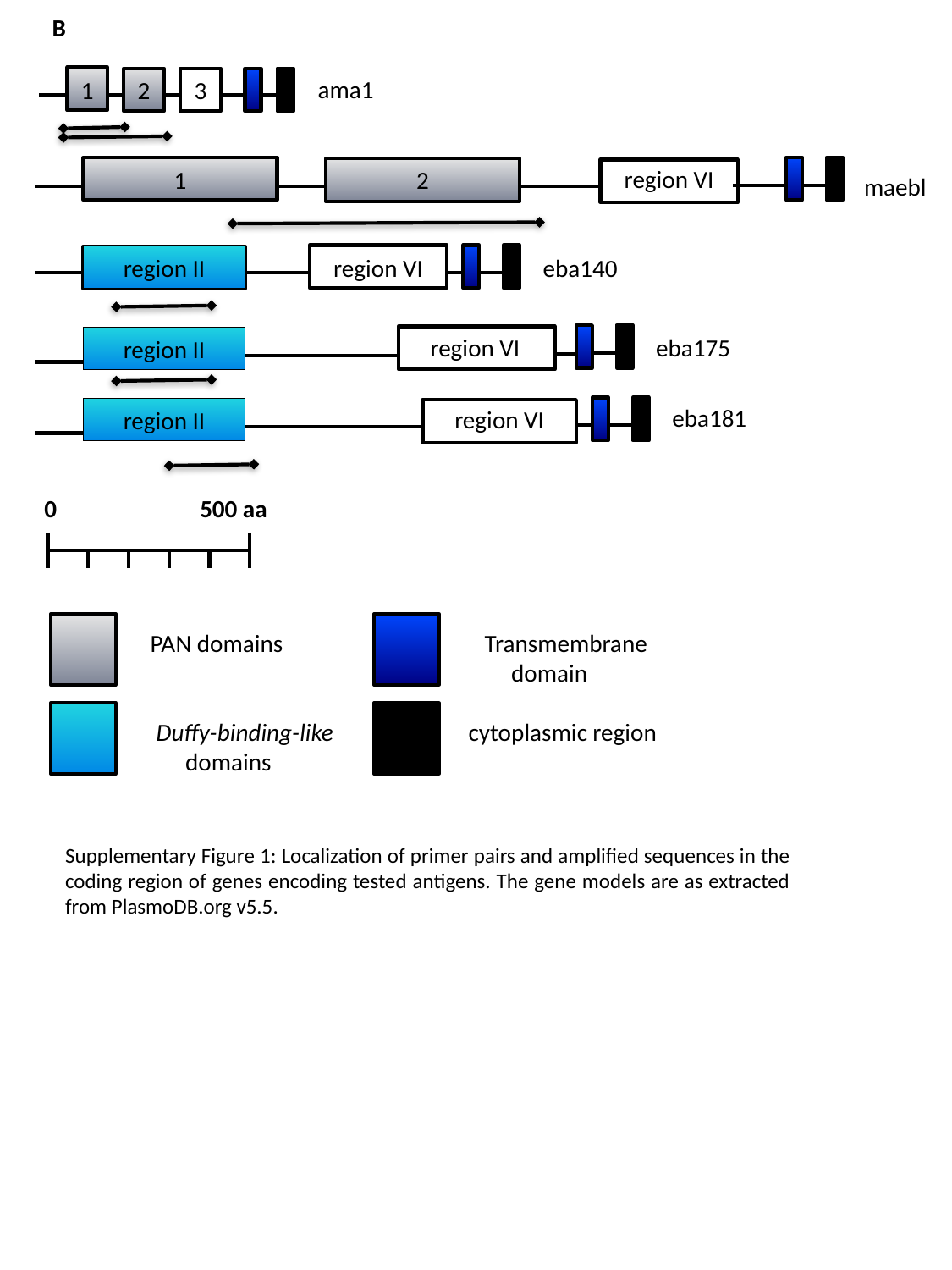

B
ama1
1
2
3
region VI
1
2
maebl
region II
region VI
eba140
region II
region VI
eba175
eba181
region VI
region II
0
500 aa
PAN domains
Transmembrane domain
Duffy-binding-like domains
cytoplasmic region
Supplementary Figure 1: Localization of primer pairs and amplified sequences in the coding region of genes encoding tested antigens. The gene models are as extracted from PlasmoDB.org v5.5.

## Slide 3
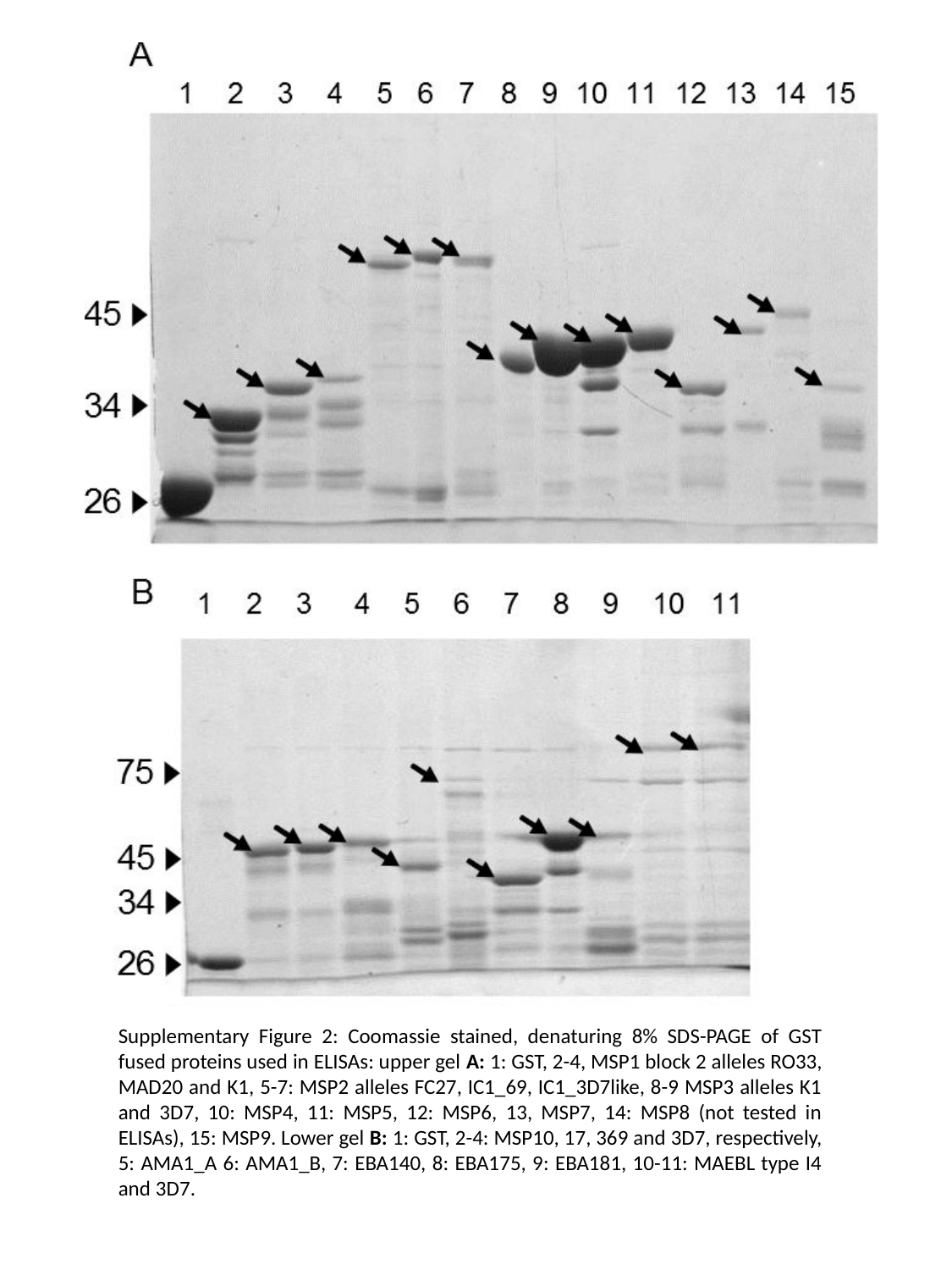

Supplementary Figure 2: Coomassie stained, denaturing 8% SDS-PAGE of GST fused proteins used in ELISAs: upper gel A: 1: GST, 2-4, MSP1 block 2 alleles RO33, MAD20 and K1, 5-7: MSP2 alleles FC27, IC1_69, IC1_3D7like, 8-9 MSP3 alleles K1 and 3D7, 10: MSP4, 11: MSP5, 12: MSP6, 13, MSP7, 14: MSP8 (not tested in ELISAs), 15: MSP9. Lower gel B: 1: GST, 2-4: MSP10, 17, 369 and 3D7, respectively, 5: AMA1_A 6: AMA1_B, 7: EBA140, 8: EBA175, 9: EBA181, 10-11: MAEBL type I4 and 3D7.

## Slide 4
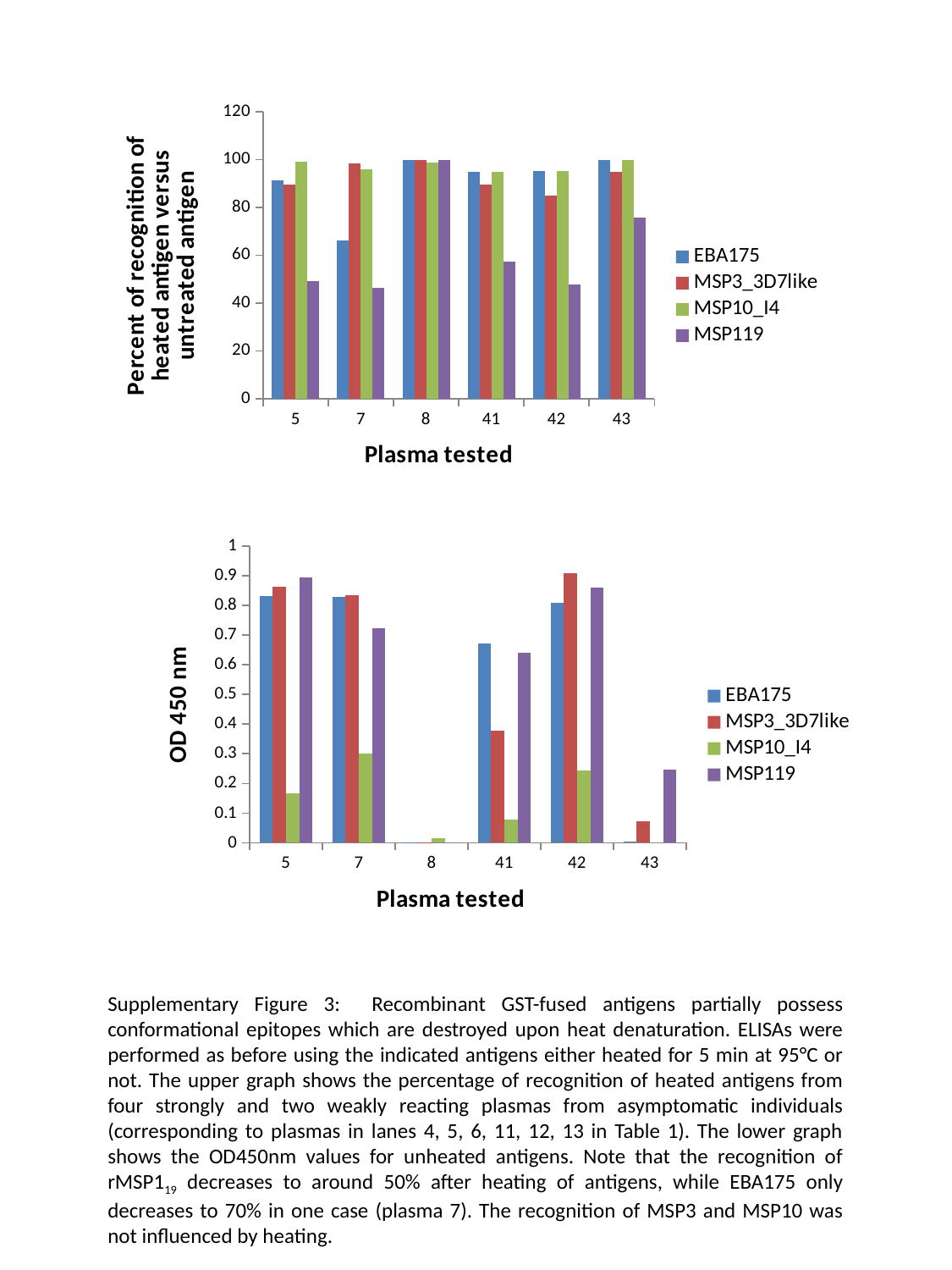

### Chart
| Category | EBA175 | MSP3_3D7like | MSP10_I4 | MSP119 |
|---|---|---|---|---|
| 5 | 91.30000000000001 | 89.4 | 99.3 | 49.3 |
| 7 | 66.30000000000001 | 98.3 | 96.0 | 46.50000000000001 |
| 8 | 99.8 | 100.0 | 98.9 | 100.0 |
| 41 | 94.80000000000001 | 89.7 | 95.0 | 57.5 |
| 42 | 95.2 | 84.9 | 95.2 | 47.8 |
| 43 | 99.7 | 94.8 | 100.0 | 75.8 |
### Chart
| Category | EBA175 | MSP3_3D7like | MSP10_I4 | MSP119 |
|---|---|---|---|---|
| 5 | 0.8310000000000003 | 0.8620000000000003 | 0.166 | 0.895 |
| 7 | 0.8280000000000003 | 0.8330000000000003 | 0.3020000000000002 | 0.7220000000000003 |
| 8 | 0.0019999999999999983 | 0.0010000000000000015 | 0.017 | 0.0 |
| 41 | 0.6730000000000003 | 0.37900000000000017 | 0.07700000000000001 | 0.6400000000000003 |
| 42 | 0.8079999999999999 | 0.909 | 0.2450000000000001 | 0.8610000000000003 |
| 43 | 0.0030000000000000022 | 0.072 | 0.0 | 0.24600000000000008 |Supplementary Figure 3: Recombinant GST-fused antigens partially possess conformational epitopes which are destroyed upon heat denaturation. ELISAs were performed as before using the indicated antigens either heated for 5 min at 95°C or not. The upper graph shows the percentage of recognition of heated antigens from four strongly and two weakly reacting plasmas from asymptomatic individuals (corresponding to plasmas in lanes 4, 5, 6, 11, 12, 13 in Table 1). The lower graph shows the OD450nm values for unheated antigens. Note that the recognition of rMSP119 decreases to around 50% after heating of antigens, while EBA175 only decreases to 70% in one case (plasma 7). The recognition of MSP3 and MSP10 was not influenced by heating.

## Slide 5
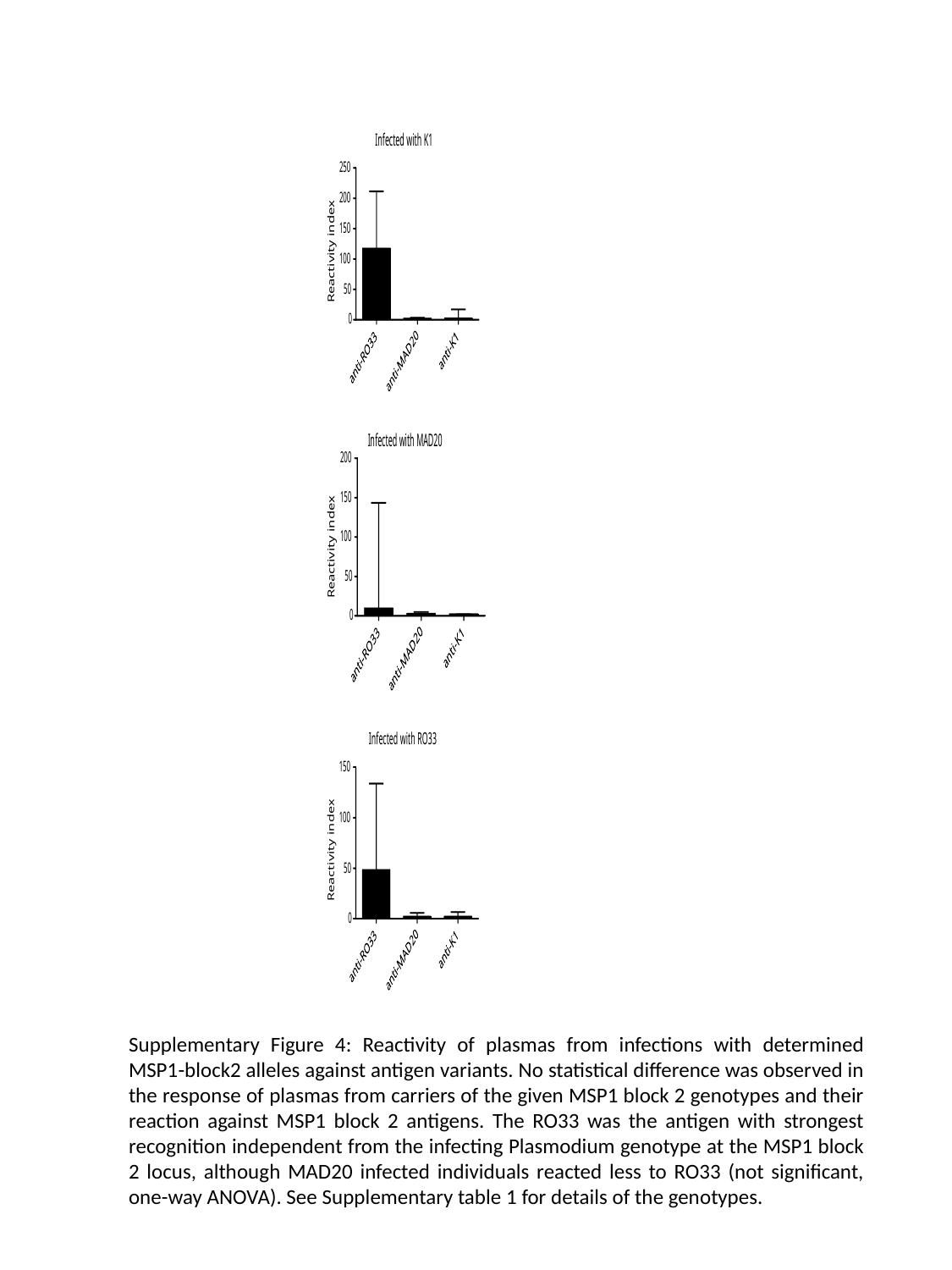

Supplementary Figure 4: Reactivity of plasmas from infections with determined MSP1-block2 alleles against antigen variants. No statistical difference was observed in the response of plasmas from carriers of the given MSP1 block 2 genotypes and their reaction against MSP1 block 2 antigens. The RO33 was the antigen with strongest recognition independent from the infecting Plasmodium genotype at the MSP1 block 2 locus, although MAD20 infected individuals reacted less to RO33 (not significant, one-way ANOVA). See Supplementary table 1 for details of the genotypes.

## Slide 6
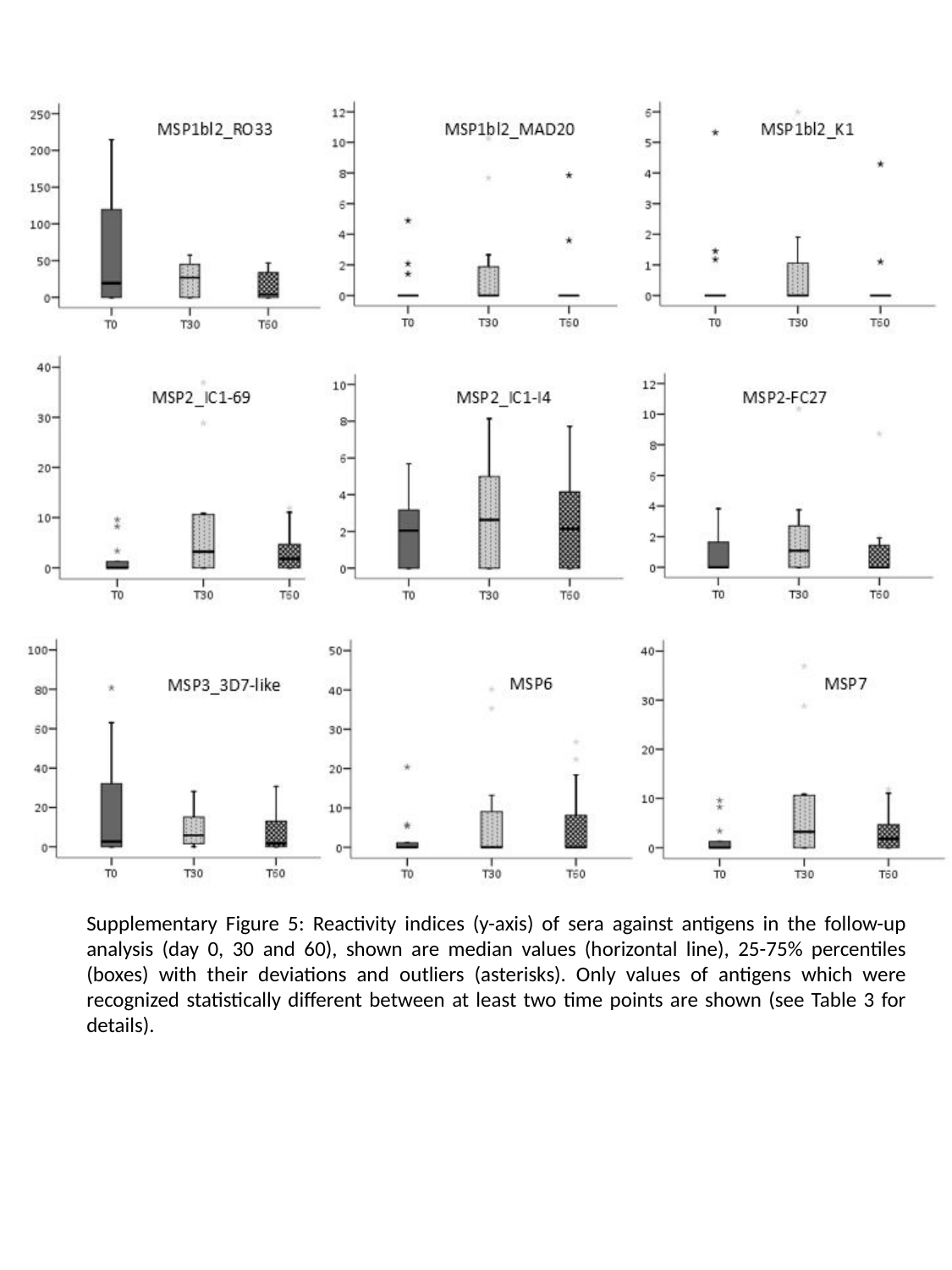

Supplementary Figure 5: Reactivity indices (y-axis) of sera against antigens in the follow-up analysis (day 0, 30 and 60), shown are median values (horizontal line), 25-75% percentiles (boxes) with their deviations and outliers (asterisks). Only values of antigens which were recognized statistically different between at least two time points are shown (see Table 3 for details).
